# Prevalence, acquisition, and risk factors of carbapenem-resistant enterobacterales colonization in long-term care hospitals: A prospective active surveillance study

**DOI:** 10.1371/journal.pone.0358973

**Published:** 2026-09-28

**Authors:** Hyejin Hwang, Sunjoo Kim

**Affiliations:** 1 Gyeongsangnam-do Center for Infectious Disease Control and Prevention, Changwon, Republic of Korea; 2 Department of Laboratory Medicine, GC Labs, Yangsan, Republic of Korea; Nitte University, INDIA

## Abstract

**Background:**

Carbapenem-resistant Enterobacterales (CRE) represent a critical threat to patient safety, particularly in long-term care hospitals (LTCHs) where elderly patients with multiple comorbidities are concentrated.

**Objective:**

To analyze asymptomatic CRE colonization, acquisition and risk factors in LTCHs prospectively.

**Methods:**

A prospective active surveillance study was conducted on 140 patients across four LTCHs in Korea from November 2023 to December 2024. Rectal swab cultures were performed weekly for up to six weeks and at three months to isolate CRE. Prevalence, acquisition rate, time to detection, and risk factors for CRE colonization were analyzed. Kaplan–Meier curves were used to compare acquisition patterns according to patient type, and Cox proportional-hazard models were applied to identify risk factors among patients who were CRE-negative at baseline.

**Results:**

The overall prevalence of asymptomatic CRE colonization was 24.3% (34/140). The colonization rate was higher among newly admitted patients (30.8%) compared to current inpatients (21.8%). Carbapenemase-producing Enterobacterales (CPE) accounted for 80.0% of CRE isolates, with KPC being predominant. Among initially negative patients, 8.6% acquired CRE during hospitalization. Kaplan–Meier analysis revealed a dynamic pattern: current inpatients showed higher acquisition rates early on, while newly admitted patients showed higher cumulative rates after three weeks. Key risk factors included invasive device use within the past year (hazard ratio, HR: 9.83) and prior room sharing with CRE patients (HR: 5.01).

**Conclusions:**

LTCHs serve as favorable environments for both CRE importation and intra-facility transmission. Strict adherence to standard precautions, targeted screening of high-risk individuals, and prompt cohorting or isolation of CRE-positive patients are essential for effective control. These findings provide prospective evidence supporting strengthened surveillance and infection prevention strategies in long-term care settings.

## Introduction

Carbapenem-resistant Enterobacterales (CRE) are recognized as one of the most critical antimicrobial-resistant pathogens worldwide and are associated with high mortality and limited treatment options [1–4]. The continuous increase in the prevalence and incidence of CRE requires awareness of its serious consequences and actions to prevent spreading of this threatening bug [2–5]. The World Health Organization has identified carbapenem-resistant Enterobacterales as critical priority pathogens, highlighting the urgent need for improved surveillance and infection prevention strategies worldwide. In a systematic review of carbapenem-resistant *Klebsiella pneumoniae* colonization, the pooled prevalence and pooled incidence were reported to be 5.43% and 22.3%, respectively [6]. The prevalence of CRE colonization (6.9%) is highest in Asia [7], which is the most populated continent. In Korea, CRE has been designated as a nationally notifiable infectious disease since 2017, and the number of reported cases has continued to increase steadily [8,9].

In Korea, long-term care hospitals (LTCHs) are hospital-based inpatient facilities that provide continuous medical care for elderly patients with multiple comorbidities. Of particular concern is the sharp rise in CRE in LTCHs, which cause prolonged hospitalization and worse outcomes for elderly patients [7,10]. These facilities are an ideal environment for silent dissemination of multi-drug-resistant organisms (MDROs) because of frequent patient–caregiver contact, substantial nursing dependency, high-density rooms, previous use of invasive devices, and repeated antibiotic exposure [11,12].

Active surveillance culture is regarded as a cornerstone strategy for preventing CRE transmission through early detection of unrecognized carriers and timely implementation of contact precautions [13,14]. However, in many Korean LTCHs, systematic surveillance remains difficult because of a shortage of infection control personnel, inadequate isolation infrastructure, lack of experience and knowledge of infection control, and insufficient reimbursement for screening tests [11]. Consequently, unrecognized CRE carriers may serve as persistent reservoirs for intra- and inter-facility spread via patient transfer [11,15].

Although numerous studies have evaluated CRE epidemiology in acute care hospitals [4,16], prospective data focusing on asymptomatic CRE colonization and acquisition in LTCHs remain limited, and many previous investigations were point-prevalence studies [11,12,17,18].

Therefore, this study prospectively evaluated unrecognized CRE colonization using repeated active surveillance cultures in patients admitted to LTCHs. The objectives were to determine the prevalence, acquisition rate, time to acquisition, and risk factors of CRE colonization and to provide practical evidence for infection control strategies in LTCHs.

## Methods

### Study design and participants

This prospective multicenter active surveillance study was conducted in four long-term care hospitals (LTCHs) located in Gyeongnam Province, Republic of Korea, between November 2023 and December 2024. The participating LTCHs were inpatient healthcare facilities providing long-term medical and nursing care for elderly patients with chronic illnesses, functional dependency, and multiple comorbidities. Active surveillance cultures were performed to evaluate asymptomatic CRE colonization, acquisition, and associated risk factors among newly admitted patients and current inpatients. Both newly admitted patients (≤2 days after admission) and current inpatients (>2 days after admission) were eligible. This study was approved by Gyeongsang National University Changwon Hospital IRB (2023-06-010). Written informed consent was obtained either from the patients or their legal representatives according to the IRB guideline.

Among 1,179 patients admitted during the study period, 26 patients with documented CRE colonization or infection within the previous year were excluded. Of the remaining 1,153 eligible patients, 140 patients who voluntarily provided written informed consent were consecutively enrolled during the study period. Because this study was conducted as a prospective feasibility-based active surveillance study in real-world LTCH settings, a formal statistical sample size calculation was not performed prior to enrollment (Fig 1). This study primarily focused on asymptomatic CRE colonization identified through active surveillance cultures in long-term care hospitals. Eligible participants were individuals without documented CRE positivity within one year prior to the initial surveillance culture. The presence of clinical symptoms at the time of the initial surveillance culture was assessed and included in the risk factor analysis. However, systematic longitudinal clinical follow-up for subsequent symptomatic CRE infection among colonized patients was not performed during the study period.

**Fig 1:**
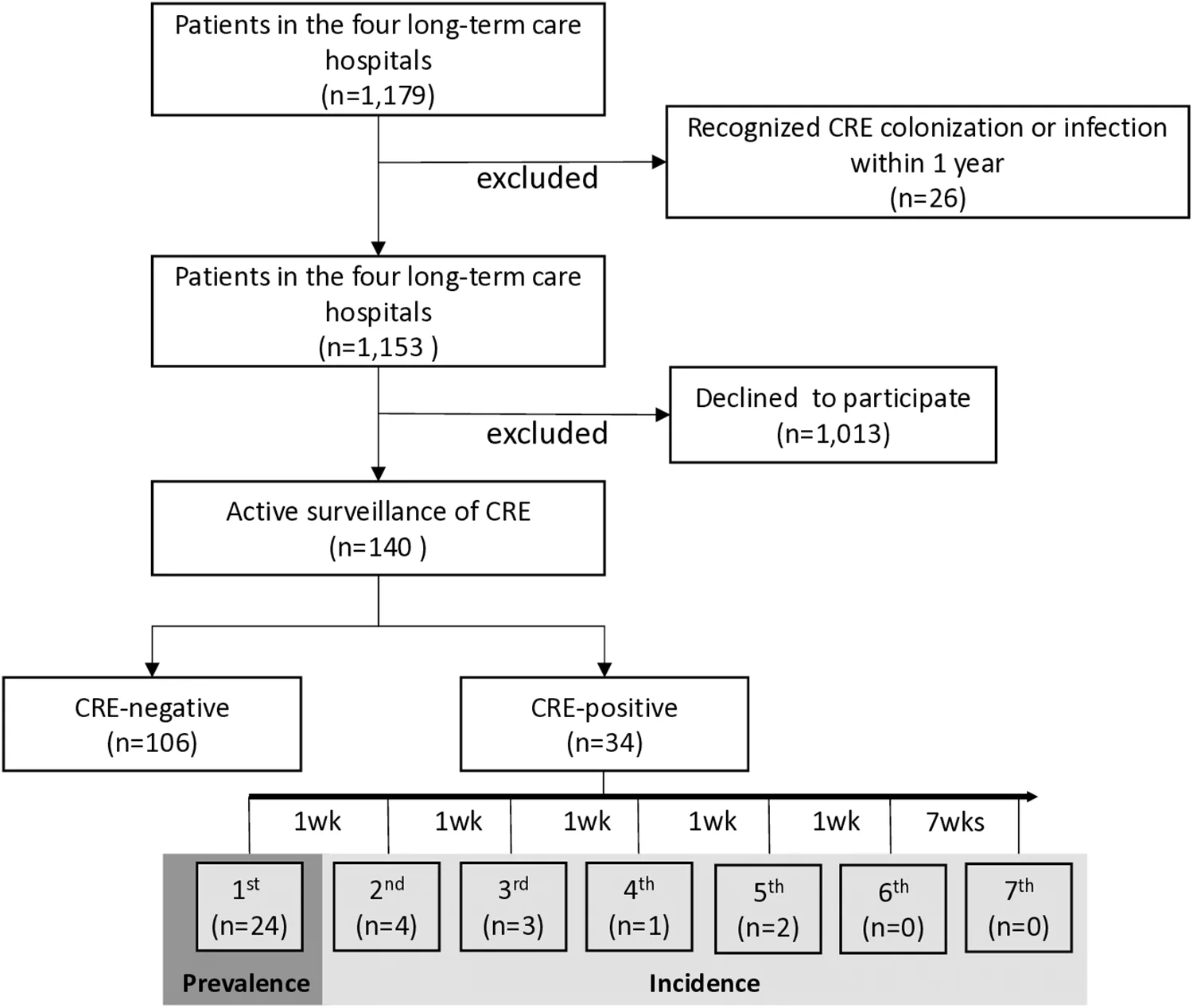
Flow diagram of patient enrollment and active surveillance.

### Active surveillance and microbiological testing

A total of 140 participants were enrolled during the study period, including both newly admitted patients and current inpatients at the time of study enrollment. The patients who did not submit the written consent were excluded. Participants were prospectively followed for up to six weeks after enrollment, and additional newly admitted patients were not continuously added during the follow-up period. Rectal swab surveillance cultures were obtained weekly for up to six consecutive weeks in accordance with international CRE screening recommendations [9,14]. Participants in whom CRE was newly detected during follow-up surveillance were excluded from subsequent surveillance cultures, and follow-up rectal swab cultures were thereafter performed only among patients who remained CRE-negative at the previous surveillance round. Accordingly, the number of participants undergoing weekly surveillance gradually decreased over time because of newly detected CRE positivity, discharge, or death from follow-up. In patients who remained CRE-negative throughout the surveillance period, an additional follow-up rectal swab culture was performed at three months (Fig 1). The rectal swab samples were cultured using MacConkey agar (Asan Pharmaceutical, Seoul, Korea) and ChromID CARBA agar (bioMerieux, Marcy l´Etoile, France). Bacteria were identified using matrix-assisted laser desorption ionization–time-of-flight mass spectrometry (bioMerieux) and antimicrobial susceptibility was tested using the VITEK-2 system (bioMerieux). CRE was defined as Enterobacterales resistant to at least one carbapenem antibiotic (imipenem, meropenem, or ertapenem) according to international and national surveillance definitions [2,3,9,19]. Carbapenemase production was confirmed by a commercial immunochromatographic assay using NG Test CARBA5 (NG-Biotech, Guipray, France)

### Data collection and statistical analysis

Clinical data were extracted from electronic medical records and included age, sex, length of hospital stay, other hospital admission, intensive care unit (ICU) admission, invasive device use, residence in a nursing home within the previous year, functional status, underlying diseases, prior MDRO colonization, antibiotic exposure within the past three months, and history of room sharing with patients known to have CRE colonization or infection within the previous year [12,16,17].

Baseline characteristics of the study participants were summarized using counts and percentages for categorical variables and means with standard deviations for continuous variables. During the follow-up period, CRE acquisition rates were visualized using Kaplan–Meier curves and compared between newly admitted patients and current inpatients using the log-rank test. In addition, to explore potential risk factors for CRE acquisition, a Cox proportional-hazard model was applied among patients who were CRE-negative at initial screening, using CRE acquisition events occurring during the follow-up period of up to 42 days. The associations were expressed as hazard ratios (HRs) with 95% confidence intervals (CIs). All statistical analyses were performed using R software (version 4.4.2; R Foundation for Statistical Computing), and an α level of 0.05 was considered statistically significant.

## Results

A total of 140 patients were enrolled: 39 were newly admitted and 101 were current inpatients (Supplementary Table 1). The overall prevalence of asymptomatic CRE colonization was 24.3% (n = 34/140). The colonization rate was higher among newly admitted patients than among current inpatients (30.8% vs. 21.8%, respectively; *p* = 0.25) (Table 1A)**.**

**Table 1:** Results of active surveillance of carbapenem-resistant Enterobacterales (CRE).

A. CRE positivity.				
Category	Negative N (%)	Positive N (%)	OR (CI)	*p-* value
Total (n=140)	106 (75.7)	34 (24.3)	1.6 (0.72-3.54)	0.25
Newly admitted patients (n=39)	27 (69.2)	12 (30.8)		
Current inpatients (n=101)	79 (78.2)	22 (21.8)		
**B. Pathogens of CRE isolates (multiple responses allowed).**				
Category			N (%)	
*Klebsiella pneumoniae*			26 (74.3)	
*Escherichia coli*			9 (25.7)	
**C. CPE among CRE isolates.**				
Category			N (%)	
Non-CPE			7 (20.0)	
CPE			28 (80.0)	
KPC			27 (96.4)	
NDM			1 (3.6)	
**D. Surveillance cycle in which CRE was first detected.**				
Surveillance round	Total N (%)	Newly admitted patients† N (%)	Current inpatients‡ N (%)	
1^st^ round	24 (70.6)	9 (75.0)	15 (68.2)	
2^nd^ round	4 (11.8)	0 (0.0)	4 (18.2)	
3^rd^ round	3 (8.8)	1 (8.3)	2 (9.1)	
4^th^ round	1 (2.9)	1 (8.3)	0 (0.0)	
5^th^ round	2 (5.9)	1 (8.3)	1 (4.5)	

†Percentage among CRE-positive newly admitted patients: (each round’s CRE-positive new patients ÷ total CRE-positive new patients) ×100.

‡Percentage among CRE-positive current inpatients: (each round’s CRE-positive current inpatients ÷ total CRE-positive current inpatients) ×100.

Abbreviations: OR, odds ratio; CI, confidence interval; CPE, carbapenemase-producing Enterobacterales; KPC, Klebsiella pneumoniae carbapenemase; NDM, New Delhi metallo-β-lactamase.

Among the 34 CRE-positive patients, a total of 35 CRE isolates were identified because one patient simultaneously harbored both *Klebsiella pneumoniae* and *Escherichia coli.* Of the 35 isolates, 26 (74.3%) were *Klebsiella pneumoniae* and 9 (25.7%) were *Escherichia coli* (Table 1B).

Carbapenemase production was confirmed in 28 of 35 CRE isolates (80.0%): 27 isolates produced KPC and one isolate produced NDM. The remaining seven isolates were classified as non-CP-CRE because no carbapenemase was detected by the CARBA5 assay. Further molecular analyses to identify non-carbapenemase resistance mechanisms, such as ESBL or AmpC production combined with porin loss, were not performed (Table 1C).

Most CRE-positive patients (70.6%) were detected as positive at the first surveillance test, indicating colonization at or near the time of admission (Table 1D).

Kaplan–Meier curves comparing cumulative CRE acquisition according to patient type (newly admitted patients vs. current inpatients) showed that during the early follow-up period (within the first three weeks), current inpatients exhibited a higher CRE acquisition rate. However, this pattern was reversed thereafter, with newly admitted patients demonstrating a relatively higher cumulative acquisition rate beyond three weeks of follow-up (Fig 2). Although the overall difference between the two groups was not statistically significant (log-rank *p* = 0.68), the delayed increase in cumulative CRE acquisition among newly admitted patients suggests that ongoing exposure within LTCH environments may contribute to progressive CRE acquisition during hospitalization.

**Fig 2:**
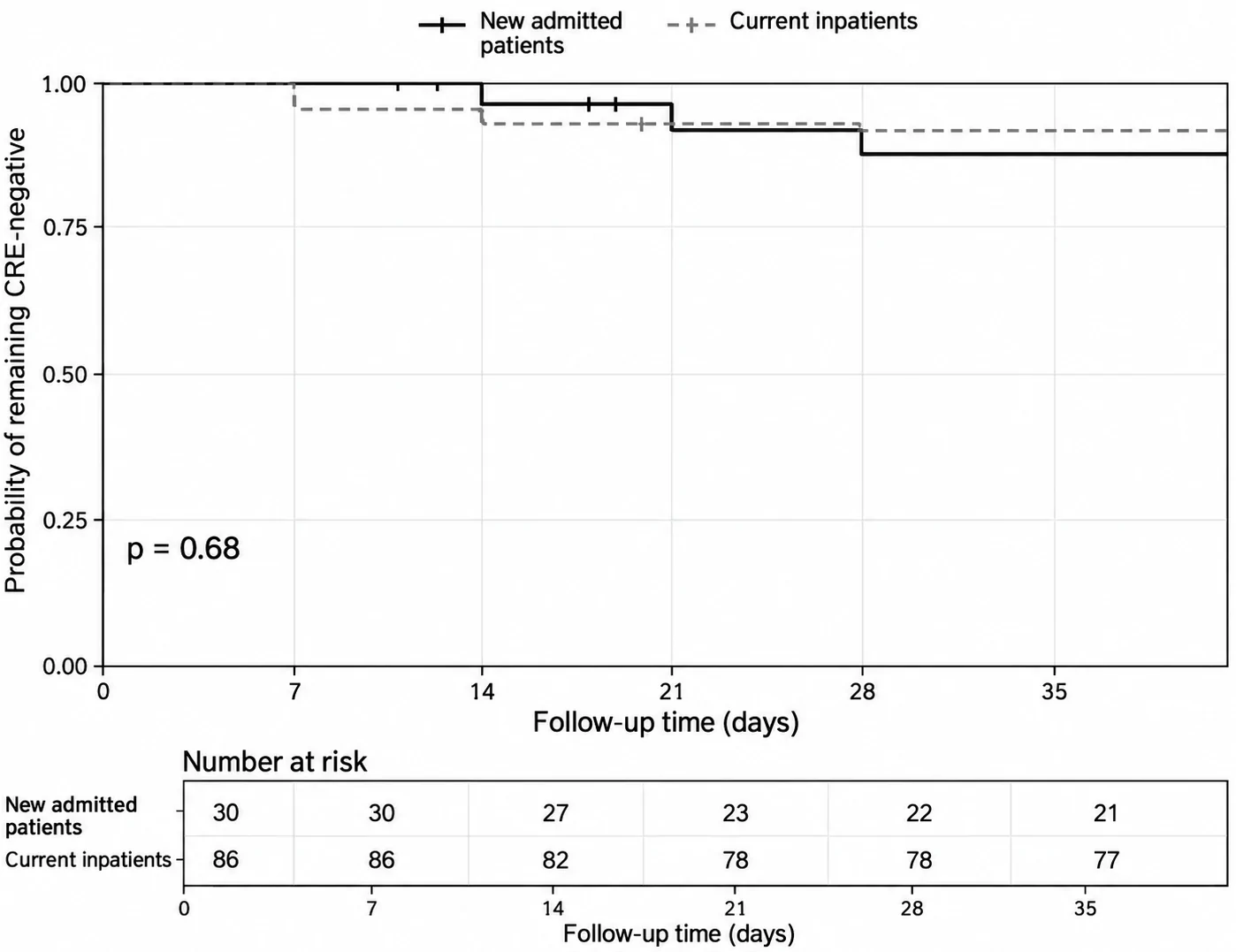
Kaplan-Meier curves showing cumulative incidence of carbapenem-resistant Enterobacterales acquisition in newly admitted patients and current in patients.

In the Cox proportional-hazard survival analysis, a history of invasive device use in the previous year was significantly associated with an increased risk of CRE acquisition (hazard ratio [HR], 9.83; 95% confidence interval [CI], 1.23–78.63; *p* = 0.031). In addition, prior room sharing with patients with CRE colonization or CRE infection in the previous year was also associated with an increased risk of CRE acquisition (HR, 5.01; 95% CI, 1.01–24.88; *p* = 0.049) (Table 2).

**Table 2:** Univariate survival analysis of risk factors for carbapenem-resistant Enterobacterales (CRE) acquisition.

Variable	Person-time	CRE *N (%)*	HR (95% CI)	*p-* value
**Patient type**				
Newly admitted patients	2,112	3 (10.0)	1.00	0.712
Current inpatients	6,893	7 (8.1)	0.78 (0.20-3.00)	
**Sex**				
Male	2,985	6 (14.3)	1.00	0.102
Female	6,020	4 (5.4)	0.35 (0.10-1.23)	
**Age (years)**				
≤59	1,520	1 (5.3)	1.00	0.164
60-69	717	4 (36.4)	7.10 (0.79-63.53)	
70-79	1,430	2 (11.1)	2.01 (0.18-22.15)	
≥80	5,338	3 (4.4)	0.84 (0.09-8.10)	
**Length of hospital stay**				
1-30 days	2,674	3 (7.9)	1.00	0.931
≥ 31 days	6.331	7 (9.0)	1.06 (0.27-4.11)	
**Other hospital admission within 1 year**				
No	4,385	4 (7.4)	1.00	0.594
Yes	4,528	6 (9.8)	1.41 (0.40-5.00)	
Unknown	92	0 (0.0)	N/A	
**ICU admission within 1 year**				
No	7,459	7 (7.4)	1.00	0.179
Yes	1,161	3 (17.6)	2.53 (0.65-9.80)	
Unknown	385	0 (0.0)	N/A	
**Invasive device usage within 1 year**				
No	4,703	1 (1.7)	1.00	0.031
Yes	3,730	8 (16.3)	9.38 (1.23-78.63)	
Unknown	572	1 (12.5)	7.80 (0.49-124.69)	
**Current invasive device**				
No	5,545	5 (7.0)	1.00	0.480
Yes	3,460	5 (11.1)	1.56 (0.45-5.40)	
**Prior CRE colonization/infection within 1 year**				
No	7,127	6 (6.8)	1.00	0.193
Yes	211	1 (25.0)	4.10 (0.49-34.29)	
Unknown	1,667	3 (12.5)	1.91 (0.48-7.65)	
**Other MDRO infection within 1 year**				
No	7,142	6 (6.7)	1.00	0.474
Yes	503	1 (14.3)	2.17 (0.26-18.04)	
Unknown	1,360	3 (15.0)	2.32 (0.58-9.30)	
**Antibiotic exposure within 3 months**				
No	5,728	5(6.8)	1.00	0.114
Yes	2,031	5 (17.9)	2.72 (0.79-9.39)	
Unknown	1,246	0 (0.0)	N/A	
**Room sharing with CRE-positive patient within 1 year**				
No	6,963	6 (7.0)	1.00	0.049
Yes	330	2 (33.3)	5.01 (1.01-24.88)	
Unknown	1,712	2 (8.3)	1.26 (0.25-6.25)	
**Residence in nursing home within 1 year**				
No	3,712	6 (11.5)	1.00	0.284
Yes	5,293	4 (6.2)	0.50 (0.14-1.78)	
**Presence of current symptoms**§				
No	7,669	8 (8.2)	1.00	0.688
Yes	1,336	2 (11.1)	1.37 (0.29-6.47)	
**Underlying diseases**				
No	1,164	2 (13.3)	1.00	0.534
Yes	7,841	8 (7.9)	0.61 (0.13-2.88)	
**Mobility state**				
Independent	1,044	1 (7.1)	1.00	0.873
Partially dependent	3,430	4 (9.1)	1.26 (0.14-11.24)	
Totally dependent	4,531	5 (8.6)	1.19 (0.14-10.20)	
**Level of consciousness**				
Alert	7,838	8 (8.1)	1.00	0.879
Drowsy	1,127	1 (6.7)	0.85 (0.11-6.81)	
Stupor/semi-coma	40	1 (50.0)	6.68 (0.83-53.64)	

Abbreviations: HR, hazard ratio; CI, confidence interval; ICU, intensive care unit; MDRO, multi-drug-resistant organism.

§Current symptoms were defined as conditions present at the time of the first active surveillance culture and included copious respiratory secretions, diarrhea, pressure ulcers, and incontinence.

## Discussion

In this prospective active surveillance study, we identified a substantial burden of CRE colonization in LTCHs. Notably, CRE colonization was detected in 30.8% of newly admitted patients, indicating that a considerable proportion of CRE burden in LTCHs is attributable to importation at the time of admission rather than in-hospital acquisition alone. This finding is consistent with previous studies reporting that LTCHs frequently receive patients already colonized with CRE from acute care hospitals or other healthcare facilities, thereby serving as amplification settings for regional CRE dissemination [7,11,12].

The observed colonization prevalence (24.3%) in our study falls within the upper range of previously reported CRE carriage rates in long-term care settings, which have varied widely from approximately 5% to over 20% depending on geographic region, patient population, and surveillance intensity [10–12]. Several studies have highlighted that reliance on clinical cultures alone substantially underestimates the true burden of CRE, as the majority of cases are identified as asymptomatic colonization rather than active infection [8,11,15]. Our findings further support the value of active surveillance cultures in LTCH settings, as a substantial proportion of CRE colonization was identified in asymptomatic individuals who would likely not have been detected through routine clinical testing alone. Active surveillance may therefore play an important role in identifying hidden reservoirs of CRE colonization and enabling earlier implementation of infection prevention and control measures [1,5,20].. The predominance of CPE (80.0%) is particularly concerning. Similar high proportions of CPE have been previously reported in LTCHs [10,12], and plasmid-mediated carbapenemase genes, such as KPC and NDM, facilitate rapid horizontal transmission, especially for *K. pneumoniae* [4]. In Spain, 9.3% out of 760 patients were colonized with carbapenemase-producing bacteria in long-term care facilities (LTCFs) [12]. In Korea, among 481 patients, CPE positivity following exposure within LCTFs was reported as 22.5% for current inpatients and 35.8% for newly admitted patients [10]. Recent CRE cases reported to the Korea Disease Control and Prevention Agency showed that prevalence doubled in LTCHs in Korea between 2019 and 2023 (7.0% vs. 15.1%, respectively) [8]. Among 38,405 reported cases of CRE in 2023, 94.6% were from colonization, whereas only 5.4% were from true infections [8]. In addition to CRE, other MDROs, such as methicillin-resistant *Staphylococcus aureus* and vancomycin-resistant enterococci, can be transferred from LTCHs to acute care facilities or vice versa [17]. The current CRE burden in LTCHs represents not only increased prevalence but also enhanced transmissibility.

In addition to CRE importation at admission, 8.6% of patients who were CRE-negative at baseline acquired CRE during hospitalization, representing the cumulative incidence of newly detected CRE colonization during the follow-up period. Notably, newly admitted patients demonstrated a median time to CRE detection of 21 days (range, 16–29 days), suggesting that CRE acquisition among newly admitted patients tended to occur within the early phase of hospitalization, particularly within the first month after admission. Comparable acquisition patterns have been reported in transfer cohorts and surveillance studies. In an emergency ICU, a CRE acquisition rate of 2.6% and acquisition duration of 10 days were documented in a tertiary-care hospital in Korea [16]. Furthermore, active surveillance of CRE showed 15.5% colonization and 28.3% acquisition, with a median acquisition time of 12 days, in a university hospital in Thailand [21]. These findings indicate that LTCHs function as dynamic transmission environments rather than passive reservoirs.

Kaplan–Meier analysis revealed that the risk of CRE acquisition varied over time according to patient type. Current inpatients exhibited higher acquisition rates during the early follow-up period, whereas newly admitted patients demonstrated a higher cumulative acquisition risk after three weeks (Fig 2). This crossing pattern suggests that CRE transmission in LTCHs is not time-invariant but reflects a dynamic interplay between prior exposure among long-stay patients and progressive accumulation of ward-level exposure among newly admitted patients.

From an epidemiologic perspective, long-stay patients may act as a reservoir, defined as a population in which a pathogen is maintained and from which ongoing transmission can occur. In contrast, newly admitted patients—who are initially CRE-negative—represent a susceptible population, and the early hospitalization period may constitute a susceptible phase during which exposure within the ward can result in negative-to-positive conversion. Thus, CRE transmission in LTCHs may be understood as an interaction between a persistent reservoir and a continuously replenished susceptible population.

This study conducted longitudinal follow-up among patients who were CRE-negative at baseline, allowing simultaneous evaluation of risk factors and temporal patterns of CRE acquisition in LTCHs. In Cox proportional-hazard analysis, a history of invasive device use within the previous year was identified as the strongest independent risk factor for CRE acquisition. This finding is consistent with previous reports indicating that invasive medical devices may facilitate CRE transmission through increased patient contact, greater opportunities for cross-transmission during medical and caregiving procedures, and associated antimicrobial exposure [1,11,12]. In addition, prior room sharing with patients colonized or infected with CRE was associated with an increased risk of acquisition, underscoring the importance of contact-based exposure management and patient cohorting at the room level in LTCH settings [5,11,22].

These findings have important implications for infection prevention strategies in LTCHs. Strict adherence to standard precautions for all patients should form the foundation of CRE prevention and control [2,3,5,14]. Although active surveillance cultures may improve the detection of unrecognized CRE colonization, we do not advocate universal routine screening for all patients in LTCH settings. Given the practical limitations of many LTCHs, including limited staffing, isolation capacity, and financial resources, targeted screening of high-risk individuals—such as those with recent invasive device use or documented contact with CRE-positive patients—may represent a more feasible, realistic, and cost-effective approach consistent with international recommendations [13,21].

Although policies for active surveillance of CRE in LTCHs have been established, active implementation is not yet available in Korea due to a critical shortage of isolation rooms, chronic understaffing of nursing personnel, and substantial financial burdens associated with caregiving and surveillance testing in the real world. The positive cases of CRE in our study were located in either the isolation room or a specific cohort. We provided education during the study period, especially focusing on caregivers who have the most frequent contact with patients, regarding infection control measures and to improve awareness of CRE.

This study has several limitations. First, the numbers of participating hospitals and enrolled patients were limited, which may restrict generalizability. Recruitment of participating LTCHs and patient enrollment were challenging because repeated active surveillance required substantial cooperation from healthcare personnel, caregivers, and patients in resource-limited real-world settings. In addition, because the number of CRE acquisition events was limited, multivariable Cox proportional hazards regression analysis was not performed due to concerns regarding statistical instability and model overfitting. Therefore, the identified risk factors should be interpreted cautiously, and further studies with larger sample sizes are needed to validate these findings using adequately powered multivariable analyses.

Second, detailed molecular investigations of transmission pathways were not performed. Detailed molecular analyses of non-carbapenemase resistance mechanisms, including ESBL or AmpC production combined with porin loss, were not performed, which limited our ability to fully characterize the mechanisms of carbapenem resistance among non-CPE isolates. Molecular study may provide more concrete transmission or acquisition data. Nevertheless, this prospective design, with repeated surveillance cultures, provides robust evidence of both CRE importation and in-hospital acquisition.

Third, participation was voluntary, which may have introduced selection bias among participating hospitals or patients. In addition, because participant enrollment was based on voluntary informed consent rather than formal probability-based sampling, the observed colonization prevalence may not fully represent the actual CRE burden within the participating facilities.

Fourth, detailed information regarding ongoing antibiotic therapy during the surveillance period was not collected. Because current antimicrobial treatment may influence CRE colonization dynamics by exerting selective antimicrobial pressure, future studies should incorporate time-dependent assessment of antibiotic exposure.

## Conclusions

A substantial proportion of patients were identified as positive for highly transmissible CRE, suggesting that CRE colonization may already have been present before or at the time of admission, while additional CRE acquisition may have occurred during hospitalization. To control CRE transmission in LTCH settings effectively, strict adherence to standard precautions, targeted screening of high-risk patients, prompt implementation of contact precautions for CRE-positive individuals, and sustained education of caregiving staff are essential components of infection prevention strategies. Moreover, to ensure that these efforts are not confined to individual facilities, a nationwide antimicrobial resistance control strategy that explicitly includes LTCHs, together with sustained infection control efforts by healthcare personnel, is warranted. In addition, regional collaborative strategies are needed to address inter-facility transmission and to achieve sustainable containment of CRE spread [13,22].

## Supporting information

S1 Table Baseline characteristics of 140 participants. (DOCX)
