# Supplementary material for: Prevalence, acquisition, and risk factors of carbapenem-resistant enterobacterales colonization in long-term care hospitals: A prospective active surveillance study
Source: PLoS One. 2026 Sep 28;21(9):e0358973. doi: 10.1371/journal.pone.0358973 (PMC13618942; doi:10.1371/journal.pone.0358973)
Supplement: Supplementary file 1 [file pone.0358973.s001.docx]

Supplementary Table. Baseline characteristics of 140 participants.

| Characteristics | N (%) |
| --- | --- |
| **Patient type** |  |
| Newly admitted patients | 39 (27.9) |
| Current inpatients | 101 (72.1) |
| **Sex** |  |
| Male | 51 (36.4) |
| Female | 89 (63.6) |
| **Age (years)** |  |
| ≤ 59 | 21 (15.0) |
| 60-69 | 14 (10.0) |
| 70-79 | 22 (15.7) |
| ≥ 80 | 83 (59.3) |
| **Length of hospital stay** |  |
| 1-30 days | 47 (33.6) |
| 31-90 days | 12 (8.6) |
| 91-180 days | 10 (7.1) |
| 181-365 days | 15 (10.7) |
| ≥ 366 days | 56 (40.0) |
| **Other hospital admission within 1 year** | |
| No | 63 (45.0) |
| Yes | 76 (54.3) |
| Unknown | 1 (0.7) |
| **ICU admission within 1 year** |  |
| No | 111 (79.3%) |
| Yes | 25 (17.9%) |
| Unknown | 4 (2.9% |
| **Invasive device use within 1 year** | |
| No | 67 (47.9%) |
| Yes | 67 (47.9%) |
| Unknown | 9 (6.4%) |
| **Current invasive device** |  |
| No | 81 (57.9%) |
| Yes | 59 (42.1%) |
| Foley catheter | 31 (22.1%) |
| Tracheostomy tube | 2 (1.4%) |
| Central venous catheter | 6 (4.3%) |
| Drainage catheter | 1 (0.7%) |
| Ostomy/gastrostomy | 1 (0.7%) |
| Levin tube | 10 (7.1%) |
| **Prior CRE colonization/infection within 1 year** | |
| No | 105 (75.0%) |
| Yes | 5 (3.6%) |
| Unknown | 30 (21.4%) |
| **Other MDRO infection within 1 year** | |
| No | 107 (76.4%) |
| Yes | 9 (6.4%) |
| Unknown | 24 (17.1%) |
| **Antibiotics exposure within 3 months** | |
| No | 83 (59.3%) |
| Yes | 41 (29.3%) |
| Unknown | 16 (11.4%) |
| **Room sharing with CRE patient within 1 year** | |
| No | 103 (73.6%) |
| Yes | 7 (5.0%) |
| Unknown | 30 (21.4%) |
| **Residence in nursing home within 1 year** | |
| No | 67 (47.9%) |
| Yes | 73 (52.1%) |
| **Presence of current symptoms*** | |
| No | 115 (82.1%) |
| Yes | 25 (17.9%) |
| **Underlying diseases** |  |
| No | 16 (11.4) |
| Yes | 124 (88.6%) |
| **Mobility state** |  |
| Independent | 16 (11.4%) |
| Partially dependent | 52 (37.1%) |
| Totally dependent | 72 (51.4%) |
| **Level of consciousness** |  |
| Alert | 116 (82.9%) |
| Drowsy | 21 (15.0%) |
| Stupor/semi-coma | 3 (2.1%) |
| Abbreviations: SD, standard deviation; ICU, intensive care units; CRE, carbapenem-resistant Enterobacterales; ICU, intensive care unit; MDRO, multi-drug-resistant organism.  ***** Current symptoms were defined as conditions present at the time of the first active surveillance culture and included copious respiratory secretions, diarrhea, pressure ulcers, and incontinence. | |
