# Supplementary material for: Prevalence, acquisition, and risk factors of carbapenem-resistant enterobacterales colonization in long-term care hospitals: A prospective active surveillance study
Source: PLoS One. 2026 Sep 28;21(9):e0358973. doi: 10.1371/journal.pone.0358973 (PMC13618942; doi:10.1371/journal.pone.0358973)
Supplement: Supplementary file 2 [file pone.0358973.s002.docx]

**Prevalence, Acquisition, and Risk Factors of Carbapenem-Resistant Enterobacterales Colonization in Long-Term Care Hospitals: A Prospective Active Surveillance Study**

Hyejin Hwang, RN, MSc^1^

Sunjoo Kim, MD, PhD^1,2*^

^1^Gyeongsangnam-do Center for Infectious Disease Control and Prevention, Changwon, Republic of Korea

^2^Department of Laboratory Medicine, GC Labs, Yangsan, Republic of Korea

***Corresponding Author**

E-mail: sjkim8239@hanmail.net

**Abstract**

**Background**

Carbapenem-resistant Enterobacterales (CRE) represent a critical threat to patient safety, particularly in long-term care hospitals (LTCHs) where elderly patients with multiple comorbidities are concentrated.

**Objective**

To analyze asymptomatic CRE colonization, acquisition and risk factors in LTCHs prospectively

**Methods**

A prospective active surveillance study was conducted on 140 patients across four LTCHs in Korea from September 2022 to December 2024. Rectal swab cultures were performed weekly for up to six weeks and at three months to isolate CRE. Prevalence, acquisition rate, time to detection, and risk factors for CRE colonization were analyzed. Kaplan–Meier curves were used to compare acquisition patterns according to patient type, and Cox proportional-hazard models were applied to identify risk factors among patients who were CRE-negative at baseline.

**Results**

The overall prevalence of asymptomatic CRE colonization was 24.3% (34/140). The colonization rate was higher among newly admitted patients (30.8%) compared to current inpatients (21.8%). Carbapenemase-producing Enterobacterales (CPE) accounted for 80.0% of CRE isolates, with KPC being predominant. Among initially negative patients, 8.6% acquired CRE during hospitalization. Kaplan–Meier analysis revealed a dynamic pattern: current inpatients showed higher acquisition rates early on, while newly admitted patients showed higher cumulative rates after three weeks. Key risk factors included invasive device use within the past year (hazard ratio, HR: 10.52) and prior room sharing with CRE patients (HR: 4.97).

**Conclusions**

LTCHs serve as favorable environments for both CRE importation and intra-facility transmission. Strict adherence to standard precautions, targeted screening of high-risk individuals, and prompt cohorting or isolation of CRE-positive patients are essential for

effective control. These findings provide prospective evidence supporting strengthened surveillance and infection prevention strategies in long-term care settings.

**Keywords:** Carbapenem-resistant Enterobacterales, Long-term care facilities, Active surveillance, Colonization, Antimicrobial resistance, Infection control

**Introduction**

Carbapenem-resistant Enterobacterales (CRE) are recognized as one of the most critical antimicrobial-resistant pathogens worldwide and are associated with high mortality and limited treatment options [1-4]. The continuous increase in the prevalence and incidence of CRE requires awareness of its serious consequences and actions to prevent spreading of this threatening bug [2-5]. The World Health Organization has identified carbapenem-resistant Enterobacterales as critical priority pathogens, highlighting the urgent need for improved surveillance and infection prevention strategies worldwide. In a systematic review of carbapenem-resistant *Klebsiella pneumoniae* colonization, the pooled prevalence and pooled incidence were reported to be 5.43% and 22.3%, respectively [6]. The prevalence of CRE colonization (6.9%) is highest in Asia [7], which is the most populated continent. In Korea, CRE has been designated as a nationally notifiable infectious disease since 2017, and the number of reported cases has continued to increase steadily [8, 9].

In Korea, long-term care hospitals (LTCHs) are hospital-based inpatient facilities that provide continuous medical care for elderly patients with multiple comorbidities. Of particular concern is the sharp rise in CRE in LTCHs, which cause prolonged hospitalization and worse outcomes for elderly patients [7, 10]. These facilities are an ideal environment for silent dissemination of multi-drug-resistant organisms (MDROs) because of frequent patient–caregiver contact, substantial nursing dependency, high-density rooms, previous use of invasive devices, and repeated antibiotic exposure [11, 12].

Active surveillance culture is regarded as a cornerstone strategy for preventing CRE transmission through early detection of unrecognized carriers and timely implementation of contact precautions [13, 14]. However, in many Korean LTCHs, systematic surveillance remains difficult because of a shortage of infection control personnel, inadequate isolation infrastructure, lack of experience and knowledge of infection control, and insufficient reimbursement for screening tests [11]. Consequently, unrecognized CRE carriers may serve as persistent reservoirs for intra- and inter-facility spread via patient transfer [11, 15].

Although numerous studies have evaluated CRE epidemiology in acute care hospitals [4, 16], prospective data focusing on asymptomatic CRE colonization and acquisition in LTCHs remain limited, and many previous investigations were point-prevalence studies [11, 12, 17, 18].

Therefore, this study prospectively evaluated unrecognized CRE colonization using repeated active surveillance cultures in patients admitted to LTCHs. The objectives were to determine the prevalence, acquisition rate, time to acquisition, and risk factors of CRE colonization and to provide practical evidence for infection control strategies in LTCHs.

**Methods**

**Study design and participants**

Enrollment of patients for active surveillance began on 14/11/2023, and continued through 31/12/2024 in four LTCHs in Gyeonggnam province, Korea. Both newly admitted patients (≤2 days after admission) and current inpatients (>2 days after admission) were eligible. Among 1,179 patients admitted during the study period, 26 patients with documented CRE colonization or infection within the previous year were excluded. Of the remaining 1,153 eligible patients, 140 who provided informed consent were finally enrolled (Fig. 1).

**Fig.1. Flow diagram of patient enrollment and active surveillance.**

**Active surveillance and microbiological testing**

Rectal swab surveillance cultures were obtained weekly for up to six consecutive weeks in accordance with international CRE screening recommendations [9, 14]. For patients who remained CRE-negative, an additional follow-up culture was obtained at three months (Figure 1). The rectal swab samples were cultured using MacConkey agar (Asan Pharmaceutical, Seoul, Korea) and ChromID CARBA agar (bioMerieux, Marcy l´Etoile, France). Bacteria were identified using matrix-assisted laser desorption ionization–time-of-flight mass spectrometry (bioMerieux) and antimicrobial susceptibility was tested using the VITEK-2 system (bioMerieux). CRE was defined as Enterobacterales resistant to at least one carbapenem antibiotic (imipenem, meropenem, or ertapenem) according to international and national surveillance definitions [2, 3, 9, 19]. Carbapenemase production was confirmed by a commercial immunochromatographic assay using NG Test CARBA5 (NG-Biotech, Guipray, France)

**Data collection and statistical analysis**

Clinical data were extracted from electronic medical records and included age, sex, length of hospital stay, other hospital admission, intensive care unit (ICU) admission, invasive device use, residence in a nursing home within the previous year, functional status, underlying diseases, prior MDRO colonization, antibiotic exposure within the past three months, and prior contact with CRE-positive patients [12, 16, 17].

Baseline characteristics of the study participants were summarized using counts and percentages for categorical variables and means with standard deviations for continuous variables. During the follow-up period, CRE acquisition rates were visualized using Kaplan–Meier curves and compared between newly admitted patients and current inpatients using the log-rank test. In addition, to explore potential risk factors for CRE acquisition, a Cox proportional-hazard model was applied among patients who were CRE-negative at initial screening, using CRE acquisition events occurring during the follow-up period of up to 42 days. The associations were expressed as hazard ratios (HRs) with 95% confidence intervals (CIs). All statistical analyses were performed using R software (version 4.4.2; R Foundation for Statistical Computing), and an α level of 0.05 was considered statistically significant.

**Results**

A total of 140 patients were enrolled: 39 were newly admitted and 101 were current inpatients (Supplementary Table 1). The overall prevalence of asymptomatic CRE colonization was 24.3% (n = 34/140). The colonization rate was higher among newly admitted patients than among current inpatients (30.8% vs. 21.8%, respectively; *p* = 0.25) (Table 1A)**.**

Table 1. Results of active surveillance of carbapenem-resistant Enterobacterales (CRE).

| A. CRE positivity. | | | | | | | | |
| --- | --- | --- | --- | --- | --- | --- | --- | --- |
| Category | | Negative | | Positive | OR (CI) | | | *p-* value |
|  |  | N (%) | | N (%) |  |  |  |  |
| Total (n=140) | | 106 (75.7) | | 34 (24.3) | 1.6  (0.72-3.54) | | | 0.25 |
| Newly admitted patients (n=39) | | 27 (69.2) | | 12 (30.8) |  | | |  |
| Current inpatients (n=101) | | 79 (78.2) | | 22 (21.8) |  | | |  |
|  | | |  | | |  | | |
| B. Pathogens of CRE isolates (multiple responses allowed). | | | | | | | | |
| Category | | | N (%) | | | | | |
| *Klebsiella pneumoniae* | | | 26 (74.3) | | | | | |
| *Escherichia coli* | | | 9 (25.7) | | | | | |
|  | | |  | | | | | |
|  | | |  | | | | | |
| C. CPE among CRE isolates. | | | | | | | | |
| Category | | | N (%) | | | | | |
| Non-CPE | | | 7 (20.0) | | | | | |
| CPE | | | 28 (80.0) | | | | | |
| KPC | | | 27 (96.4) | | | | | |
| NDM | | | 1 (3.6) | | | | | |
|  | | | | | | | | |
| D. Surveillance cycle in which CRE was first detected. | | | | | | | | |
| Surveillance  round | Total | | Newly admitted patients† | | | | Current inpatients‡ | |
|  | N (%) | | N (%) | | | | N (%) | |
| 1^st^ round | 24 (70.6) | | 9 (75.0) | | | | 15 (68.2) | |
| 2^nd^ round | 4 (11.8) | | 0 (0.0) | | | | 4 (18.2) | |
| 3^rd^ round | 3 (8.8) | | 1 (8.3) | | | | 2 (9.1) | |
| 4^th^ round | 1 (2.9) | | 1 (8.3) | | | | 0 (0.0) | |
| 5^th^ round | 2 (5.9) | | 1 (8.3) | | | | 1 (4.5) | |
| †Percentage among CRE-positive newly admitted patients: (each round’s CRE-positive new patients ÷ total CRE-positive new patients) ×100.  ‡Percentage among CRE-positive current inpatients: (each round’s CRE-positive current inpatients ÷ total CRE-positive current inpatients) ×100.  Abbreviations: OR, odds ratio; CI, confidence interval; CPE, carbapenemase-producing Enterobacterales; KPC, Klebsiella pneumoniae carbapenemase; NDM, New Delhi metallo-β-lactamase. | | | | | | | | |

Among the 35 CRE isolates, 26 (74.3%) were *K. pneumoniae* and 9 (25.7%) were *Escherichia coli* (Table 1B). One patient harbored these two bacteria at the same time.

Carbapenemase-producing Enterobacterales (CPE) accounted for 28 (80.0%) of the 35 CRE isolates, with KPC being the predominant carbapenemase (Table 1C).

Most CRE-positive patients (70.6%) were detected as positive at the first surveillance test, indicating colonization at or near the time of admission (Table 1D).

Kaplan–Meier curves comparing cumulative CRE acquisition according to patient type (newly admitted patients vs. current inpatients) showed that during the early follow-up period (within the first three weeks), current inpatients exhibited a higher CRE acquisition rate. However, this pattern was reversed thereafter, with newly admitted patients demonstrating a higher cumulative acquisition rate beyond three weeks of follow-up (Fig. 2).

**Fig 2. Kaplan-Meier curves showing cumulative incidence of carbapenem-resistant Enterobacterales acquisition in newly admitted patients and current in patients.**

In the Cox proportional-hazard survival analysis, a history of invasive device use in the previous year was significantly associated with an increased risk of CRE acquisition (hazard ratio [HR], 10.52; 95% confidence interval [CI], 1.32–84.11; *p* = 0.02). In addition, prior room sharing with patients with CRE colonization or CRE infection in the previous year was also associated with an increased risk of CRE acquisition (HR, 4.97; 95% CI, 1.00–24.65) (Table 2).

Table 2. Univariate survival analysis of risk factors for carbapenem-resistant Enterobacterales (CRE) acquisition.

| Variable | Person-time | CRE  N (%) | HR  (95% CI) | *p-*  value |
| --- | --- | --- | --- | --- |
| **Patient type** | | | | |
| Newly admitted patients | 1,197 | 3 (10.0) | 1.00 | 0.79 |
| Current inpatients | 3,420 | 7 (8.1) | 0.83 (0.22-3.22) |  |
| **Sex** | | | | |
| Male | 1,575 | 6 (14.3) | 1.00 | 0.11 |
| Female | 3,024 | 4 (5.4) | 0.35 (0.10-1.25) |  |
| **Age (years)** | | | | |
| ≤59 | 763 | 1 (5.3) | 1.00 | 0.04 |
| 60-69 | 364 | 4 (36.4) | 7.65 (0.85-68.51) |  |
| 70-79 | 714 | 2 (11.1) | 2.08 (0.19-22.96) |  |
| ≥80 | 2,758 | 3 (4.4) | 0.83 (0.09-7.96) |  |
| **Length of hospital stay** | | | | |
| 1-30 days | 1,806 | 5 (10.9) | 1.00 | 0.51 |
| ≥ 31 days | 2,793 | 5 (7.1) | 0.66 (0.19-2.27) |  |
| **Other hospital admission within 1 year** | | | | |
| No | 3,170 | 4 (7.4) | 1.00 | 0.80 |
| Yes | 2,387 | 6 (9.8) | 1.35 (0.38-4.80) |  |
| Unknown | 42 | 0 (0.0) | N/A |  |
| **ICU admission within 1 year** | | | | |
| No | 3,801 | 7 (7.4) | 1.00 | 0.32 |
| Yes | 630 | 4 (17.6) | 2.52 (0.65-9.74) |  |
| Unknown | 168 | 0 (0.0) | N/A |  |
| **Invasive device usage within past year** | | | | |
| No | 2,464 | 1 (1.7) | 1.00 | 0.02 |
| Yes | 1,827 | 8 (16.3) | 10.52 (1.32-84.11) |  |
| Unknown | 308 | 1 (12.5) | 7.78 (0.49-124.35) |  |
| **Current invasive device** | | | | |
| No | 2,842 | 5 (7.0) | 1.00 | 0.46 |
| Yes | 1,757 | 5 (11.1) | 1.60 (0.46-5.54) |  |
| **Prior CRE colonization/infection within 1 year** | | | | |
| No | 3,507 | 6 (6.8) | 1.00 | 0.45 |
| Yes | 140 | 1 (25.0) | 3.92 (0.47-32.59) |  |
| Unknown | 952 | 3 (12.5) | 1.80 (0.45-7.18) |  |
| **Other MDRO infection within 1 year** | | | | |
| No | 3,549 | 6 (6.7) | 1.00 | 0.51 |
| Yes | 266 | 1 (14.3) | 2.16 (0.26-17.94) |  |
| Unknown | 784 | 3 (15.0) | 2.19 (0.55-8.76) |  |
| **Antibiotic exposure within 3 months** | | | | |
| No | 2,940 | 5(6.8) | 1.00 | 0.07 |
| Yes | 1,029 | 5 (17.9) | 2.79 (0.81-9.64) |  |
| Unknown | 630 | 0 (0.0) | N/A |  |
| **Room sharing with CRE-positive patient within 1 year** | | | | |
| No | 3,430 | 6 (7.0) | 1.00 | 0.24 |
| Yes | 210 | 2 (33.3) | 4.97 (1.00-24.65) |  |
| Unknown | 959 | 2 (8.3) | 1.18 (0.23-5.83) |  |
| **Residence in nursing home within 1 year** | | | | |
| No | 2,023 | 6 (11.5) | 1.00 | 0.32 |
| Yes | 2,576 | 4 (6.2) | 0.53 (0.15-1.88) |  |
| **Presence of current symptoms**§ | | | | |
| No | 3,892 | 8 (8.2) | 1.00 | 0.70 |
| Yes | 707 | 2 (11.1) | 1.37 (0.29-6.45) |  |
| **Underlying diseases** | | | | |
| No | 1,827 | 3 (6.7) | 1.00 | 0.52 |
| Yes | 2,772 | 7 (9.9) | 1.54 (0.40-5.96) |  |
| **Mobility state** | | | | |
| Independent | 560 | 1 (7.1) | 1.00 | 0.97 |
| Partially dependent | 1,743 | 4 (9.1) | 1.28 (0.14-11.48) |  |
| Totally dependent | 2,296 | 5 (8.6) | 1.23 (0.14-10.49) |  |
| **Level of consciousness** | | | | |
| Alert | 3,941 | 8 (8.1) | 1.00 | 0.35 |
| Drowsy | 595 | 1 (6.7) | 0.84 (0.11-6.69) |  |
| Stupor/semi-coma | 63 | 1 (50.0) | 6.68 (0.83-53.64) |  |
| Abbreviations: HR, hazard ratio; CI, confidence interval; ICU, intensive care unit; MDRO, multi-drug-resistant organism.  §Current symptoms were defined as conditions present at the time of the first active surveillance culture and included copious respiratory secretions, diarrhea, pressure ulcers, and incontinence. | | | | |

**Discussion**

In this prospective active surveillance study, we identified a substantial burden of CRE colonization in LTCHs. Notably, CRE colonization was detected in 30.8% of newly admitted patients, indicating that a considerable proportion of CRE burden in LTCHs is attributable to importation at the time of admission rather than in-hospital acquisition alone. This finding is consistent with previous studies reporting that LTCHs frequently receive patients already colonized with CRE from acute care hospitals or other healthcare facilities, thereby serving as amplification settings for regional CRE dissemination [7, 11, 12].

The observed colonization prevalence in our study falls within the upper range of previously reported CRE carriage rates in long-term care settings, which have varied widely from approximately 5% to over 20% depending on geographic region, patient population, and surveillance intensity [10-12]. Several studies have highlighted that reliance on clinical cultures alone substantially underestimates the true burden of CRE, as the majority of cases are identified as asymptomatic colonization rather than active infection [8, 11, 15]. Our findings further support that LTCHs represent major reservoirs of unrecognized CRE colonization, emphasizing the limitations of symptom-based surveillance strategies [1, 5, 20]. The predominance of CPE (80.0%) is particularly concerning. Similar high proportions of CPE have been previously reported in LTCHs [10, 12], and plasmid-mediated carbapenemase genes, such as KPC and NDM, facilitate rapid horizontal transmission, especially for *K. pneumoniae* [4]. In Spain, 9.3% out of 760 patients were colonized with carbapenemase-producing bacteria in long-term care facilities (LTCFs) [12]. In Korea, among 481 patients, CPE positivity following exposure within LCTFs was reported as 22.5% for current inpatients and 35.8% for newly admitted patients [10]. Recent CRE cases reported to the Korea Disease Control and Prevention Agency showed that prevalence doubled in LTCHs in Korea between 2019 and 2023 (7.0% vs. 15.1%, respectively) [8]. Among 38,405 reported cases of CRE in 2023, 94.6% were from colonization, whereas only 5.4% were from true infections [8]. In addition to CRE, other MDROs, such as methicillin-resistant *Staphylococcus aureus* and vancomycin-resistant enterococci, can be transferred from LTCHs to acute care facilities or vice versa [17]. The current CRE burden in LTCHs represents not only increased prevalence but also enhanced transmissibility.

In addition to importation at admission, negative-to-positive conversion occurred in 8.6% of initially CRE-negative patients. Notably, newly admitted patients demonstrated a median time to CRE detection of 21 days (range, 16–29 days), suggesting that CRE acquisition among newly admitted patients tended to occur within the early phase of hospitalization, particularly within the first month after admission. Comparable acquisition patterns have been reported in transfer cohorts and surveillance studies. In an emergency ICU, a CRE acquisition rate of 2.6% and acquisition duration of 10 days were documented in a tertiary-care hospital in Korea [16]. Furthermore, active surveillance of CRE showed 15.5% colonization and 28.3% acquisition, with a median acquisition time of 12 days, in a university hospital in Thailand [21]. These findings indicate that LTCHs function as dynamic transmission environments rather than passive reservoirs.

Kaplan–Meier analysis revealed that the risk of CRE acquisition varied over time according to patient type. Current inpatients exhibited higher acquisition rates during the early follow-up period, whereas newly admitted patients demonstrated a higher cumulative acquisition risk after three weeks (Figure 2). This crossing pattern suggests that CRE transmission in LTCHs is not time-invariant but reflects a dynamic interplay between prior exposure among long-stay patients and progressive accumulation of ward-level exposure among newly admitted patients.

From an epidemiologic perspective, long-stay patients may act as a reservoir, defined as a population in which a pathogen is maintained and from which ongoing transmission can occur. In contrast, newly admitted patients—who are initially CRE-negative—represent a susceptible population, and the early hospitalization period may constitute a susceptible phase during which exposure within the ward can result in negative-to-positive conversion. Thus, CRE transmission in LTCHs may be understood as an interaction between a persistent reservoir and a continuously replenished susceptible population.

This study conducted longitudinal follow-up among patients who were CRE-negative at baseline, allowing simultaneous evaluation of risk factors and temporal patterns of CRE acquisition in LTCHs. In Cox proportional-hazard analysis, a history of invasive device use within the previous year was identified as the strongest independent risk factor for CRE acquisition. This finding is consistent with previous reports indicating that invasive medical devices may facilitate CRE transmission through increased patient contact, greater opportunities for cross-transmission during medical and caregiving procedures, and associated antimicrobial exposure [1, 11, 12]. In addition, prior room sharing with patients colonized or infected with CRE was associated with an increased risk of acquisition, underscoring the importance of contact-based exposure management and patient cohorting at the room level in LTCH settings [5, 11, 22].

These findings have important implications for infection prevention strategies in LTCHs. Rather than relying solely on time-limited or patient group-specific surveillance approaches, strict adherence to standard precautions for all patients would form the foundation of CRE control [2, 3, 5, 14]. Given the practical constraints of universal active surveillance in LTCHs, targeted screening of high-risk individuals—such as those with recent invasive device use or documented contact with CRE-positive patients—represents a realistic and effective approach consistent with international recommendations [13, 21].

Although policies for active surveillance of CRE in LTCHs have been established, active implementation is not yet available in Korea due to a critical shortage of isolation rooms, chronic understaffing of nursing personnel, and substantial financial burdens associated with caregiving and surveillance testing in the real world. The positive cases of CRE in our study were located in either the isolation room or a specific cohort. We provided education during the study period, especially focusing on caregivers who have the most frequent contact with patients, regarding infection control measures and to improve awareness of CRE.

This study has several limitations. First, the numbers of participating hospitals and enrolled patients were limited, which may restrict generalizability. Without any incentives, it was very difficult to recruit the LTCHs and to enroll the patients. Second, detailed molecular investigations of transmission pathways were not performed. Molecular study may provide more concrete transmission or acquisition data. Nevertheless, this prospective design, with repeated surveillance cultures, provides robust evidence of both CRE importation and in-hospital acquisition. Third, participation was voluntary, which may have introduced selection bias among participating hospitals or patients.

**Conclusions**

A substantial proportion of patients entered LTCHs already colonized with highly transmissible CRE, and additional acquisition occurred over the course of hospitalization. To control CRE transmission in LTCH settings effectively, strict adherence to standard precautions, targeted screening of high-risk patients, prompt implementation of contact precautions for CRE-positive individuals, and sustained education of caregiving staff are essential components of infection prevention strategies. Moreover, to ensure that these efforts are not confined to individual facilities, a nationwide antimicrobial resistance control strategy that explicitly includes LTCHs, together with sustained infection control efforts by healthcare personnel, is warranted. In addition, regional collaborative strategies are needed to address inter-facility transmission and to achieve sustainable containment of CRE spread [13, 22].

## ****Ethics approval and consent to participate****

This study was approved by Gyeongsang National University Changwon Hospital IRB (2023-06-010). Written informed consent was obtained either from the patients or their legal representatives according to the IRB guideline.

## ****Consent for publication****

Not applicable.

## ****Availability of data and materials****

Data are available from the corresponding author upon reasonable request.

## ****Competing interests****

The authors declare no competing interests.

## ****Funding****

## This study was supported by the Ministry of Trade, Industry, and Energy of Korea (grant No. RS 2024-00403563). The funders had no role in the study design, data collection and interpretation, or decision to submit the manuscript for publication.

## ****Authors’ contributions****

## Hyejin Hwang, RN, MSc, contributed to the study design, data collection, active surveillance coordination, statistical analysis, and drafting of the initial manuscript. Sunjoo Kim, MD, PhD, conceived and supervised the study, secured funding, performed the microbiological interpretation, and critically revised the intellectual content of the manuscript. The authors read and approved the final manuscript.

## ****Acknowledgements****

The authors express their sincere gratitude to the infection control teams and designated coordinators at the four participating LTCHs for their invaluable cooperation in patient recruitment, data collection, and active surveillance procedures. We also thank the staff of the Division of Clinical Microbiology, Department of Laboratory Medicine, Gyeongsang National University Changwon Hospital, for their dedicated technical support in microbiological testing and carbapenemase detection. We are grateful for the advice and technical support from ICCON (Infection Control Consulting Network) of Korea. Their contributions were essential for the successful completion of this study.

**References**

1. Chen HY, Jean SS, Lee YL, Lu MC, Ko WC, Liu PY, et al. Carbapenem-Resistant Enterobacterales in Long-Term Care Facilities: A Global and Narrative Review. Front Cell Infect Microbiol. 2021 Apr 23;11:601968. doi: 10.3389/fcimb.2021.601968. PMID: 33968793; PMCID: PMC8102866.
2. European Centre for Disease Prevention and Control. Carbapenem-resistant Enterobacterales, third update. Stockholm: ECDC; 2025. Available from: <https://www.ecdc.europa.eu/en/publications-data/carbapenem-resistant-enterobacterales-rapid-risk-assessment-third-update> (accessed 2025.12.20).
3. World Health Organization. Guidelines for the prevention and control of carbapenem-resistant Enterobacteriaceae, *Acinetobacter baumannii* and *Pseudomonas aeruginosa* in health-care facilities. Geneva: WHO; 2017. Available from: <https://www.ncbi.nlm.nih.gov/books/NBK493061/> (accessed 2025.12.20).
4. Lee SH, Kim CH, Lee HY, Park KH, Han SH. Epidemiology of Carbapenem-Resistant *Enterobacteriaceae* Bacteremia in Gyeonggi Province, Republic of Korea, between 2018 and 2021. Antibiotics (Basel). 2023 Aug 4;12(8):1286. doi: 10.3390/antibiotics12081286. PMID: 37627706; PMCID: PMC10451680.
5. **Illinois Department of Public Health.** Prevention, control, and management of carbapenem-resistant Enterobacteriaceae in long-term care facilities. Springfield (IL): Illinois Department of Public Health; 2016. Available from: https://dph.illinois.gov/content/dam/soi/en/web/idph/files/publications/management-cre-ltcf-040516.pdf (accessed 2025.12.20).
6. Tesfa T, Mitiku H, Edae M, Assefa N. Prevalence and incidence of carbapenem-resistant K. pneumoniae colonization: systematic review and meta-analysis. Syst Rev. 2022 Nov 15;11(1):240. doi: 10.1186/s13643-022-02110-3. PMID: 36380387; PMCID: PMC9667607.
7. Rodríguez-Villodres Á, Martín-Gandul C, Peñalva G, Guisado-Gil AB, Crespo-Rivas JC, Pachón-Ibáñez ME, et al, Cisneros JM. Prevalence and Risk Factors for Multidrug-Resistant Organisms Colonization in Long-Term Care Facilities Around the World: A Review. Antibiotics (Basel). 2021 Jun 7;10(6):680. doi: 10.3390/antibiotics10060680. PMID: 34200238; PMCID: PMC8228357.
8. Lee H, Lee S, Jung YH, Choi J, Park SK. Characteristics of Notified Carbapenem-resistant *Enterobacterales* Cases in the Republic of Korea, 2023. Jugan Geongang Gwa Jilbyeong. 2025 Jan 9;18(2):75-89. doi: 10.56786/PHWR.2025.18.2.2. Epub 2024 Dec 11. PMID: 41334055; PMCID: PMC12272717.
9. Korea Disease Control and Prevention Agency. 2024 Guidelines for control of healthcare-associated infection. Cheongju (KR): Korea Disease Control and Prevention Agency; 2024.
10. Park JJ, Seo YB, Lee J, Eom JS, Song W, Choi YK, et al. Positivity of Carbapenemase-producing *Enterobacteriaceae* in Patients Following Exposure within Long-term Care Facilities in Seoul, Korea. J Korean Med Sci. 2020 Sep 14;35(36):e303. doi: 10.3346/jkms.2020.35.e303. PMID: 32924341; PMCID: PMC7490199.
11. Park Y, Son E, Choe YJ, Kang CR, Roh S, Hwang YO, et al. Outbreak of carbapenem-resistant Enterobacterales at a long-term care facility in Seoul, Korea: surveillance and intervention mitigation strategies. Epidemiol Health. 2023;45:e2023057. doi: 10.4178/epih.e2023057. Epub 2023 Jun 9. PMID: 37321276; PMCID: PMC10482563. .
12. Callejón Fernández M, Madueño Alonso A, Abreu Rodríguez R, Aguirre-Jaime A, Castro Hernández MB, Ramos-Real MJ, et al. Risk factors for colonization by carbapenemase-producing bacteria in Spanish long-term care facilities: a multicentre point-prevalence study. Antimicrob Resist Infect Control. 2022 Dec 20;11(1):163. doi: 10.1186/s13756-022-01200-0. PMID: 36536455; PMCID: PMC9763085. .
13. Jenkins DR, Auckland C, Chadwick C, Dodgson AR, Enoch DA, Goldenberg SD, et al. A practical approach to screening for carbapenemase-producing Enterobacterales- views of a group of multidisciplinary experts from English hospitals. BMC Infect Dis. 2024 Apr 26;24(1):444. doi: 10.1186/s12879-024-09307-y. PMID: 38671365; PMCID: PMC11046869.
14. Magiorakos AP, Burns K, Rodríguez Baño J, Borg M, Daikos G, et al. Infection prevention and control measures and tools for the prevention of entry of carbapenem-resistant *Enterobacteriaceae* into healthcare settings: guidance from the European Centre for Disease Prevention and Control. Antimicrob Resist Infect Control. 2017 Nov 15;6:113. doi: 10.1186/s13756-017-0259-z. PMID: 29163939; PMCID: PMC5686856.
15. Spencer MD, Winglee K, Passaretti C, Earl AM, Manson AL, Mulder HP, et al. Whole Genome Sequencing detects Inter-Facility Transmission of Carbapenem-resistant Klebsiella pneumoniae. J Infect. 2019 Mar;78(3):187-199. doi: 10.1016/j.jinf.2018.11.003. Epub 2018 Nov 29. PMID: 30503842; PMCID: PMC6408229.
16. Kang JS, Yi J, Ko MK, Lee SO, Lee JE, Kim KH. Prevalence and Risk Factors of Carbapenem resistant *Enterobacteriaceae* Acquisition in an Emergency Intensive Care Unit in a Tertiary Hospital in Korea: a Case-Control Study. J Korean Med Sci. 2019 May 13;34(18):e140. doi: 10.3346/jkms.2019.34.e140. PMID: 31074254; PMCID: PMC6509365.
17. Jeong H, Kang S, Cho HJ. Prevalence of Multidrug-Resistant Organisms and Risk Factors for Carriage among Patients Transferred from Long-Term Care Facilities. Infect Chemother. 2020 Jun;52(2):183-193. doi: 10.3947/ic.2020.52.2.183. Epub 2020 May 29. PMID: 32468740; PMCID: PMC7335643.
18. Almadhoon H, Ahmad I, Lee WWY, Matthews A, Carter ER, Otter JA, et al. Prevalence of carbapenem-resistant Enterobacterales in healthcare and community settings in the UK: a systematic review and meta-analysis. J Hosp Infect. 2025 Nov;165:19-31. doi: 10.1016/j.jhin.2025.07.030. Epub 2025 Aug 20. PMID: 40846285. .
19. Centers for Disease Control and Prevention. Carbapenem-Resistant Enterobacteriaceae (CRE): November 2015 Update – CRE Toolkit. Atlanta (GA) Centers for Disease Control and Prevention;2015. Available from: https://archive.ahrq.gov/hai/patient-safety-resources/cre-toolkit/cretoolkit_0.pdf (accessed 2025.12.20).
20. Bartsch SM, Wong KF, Stokes-Cawley OJ, McKinnell JA, Cao C, Gussin GM,,et al. Knowing More of the Iceberg: How Detecting a Greater Proportion of Carbapenem-Resistant Enterobacteriaceae Carriers Influences Transmission. J Infect Dis. 2020 May 11;221(11):1782-1794. doi: 10.1093/infdis/jiz288. PMID: 31150539; PMCID: PMC7213567.
21. Wangchinda W, Thamlikitkul V, Watcharasuwanseree S, Tangkoskul T. Active Surveillance for Carbapenem-Resistant *Enterobacterales* (CRE) Colonization and Clinical Course of CRE Colonization among Hospitalized Patients at a University Hospital in Thailand. Antibiotics (Basel). 2022 Oct 13;11(10):1401. doi: 10.3390/antibiotics11101401. PMID: 36290059; PMCID: PMC9598097. .
22. Ben-David D, Masarwa S, Fallach N, Temkin E, Solter E, Carmeli Y, et al. Success of a National Intervention in Controlling Carbapenem-resistant Enterobacteriaceae in Israel's Long-term Care Facilities. Clin Infect Dis. 2019 Mar 5;68(6):964-971. doi: 10.1093/cid/ciy572. PMID: 29986007.

**Supporting information**

**S1 Fig. Flow diagram of patient enrollment and active surveillance.**

**S2 Fig. Kaplan-Meier curves showing cumulative incidence of carbapenem-resistant Enterobacterales acquisition in newly admitted patients and current in patients.**

**S1 Table. Results of active surveillance of carbapenem-resistant Enterobacterales (CRE).**

**S2 Table. Univariate survival analysis of risk factors for carbapenem-resistant Enterobacterales (CRE) acquisition.**

**S3 Supplementary Table. Baseline characteristics of 140 participants.**
