# Supplementary material for: Prevalence, acquisition, and risk factors of carbapenem-resistant enterobacterales colonization in long-term care hospitals: A prospective active surveillance study
Source: PLoS One. 2026 Sep 28;21(9):e0358973. doi: 10.1371/journal.pone.0358973 (PMC13618942; doi:10.1371/journal.pone.0358973)
Supplement: Supplementary file 3 [file pone.0358973.s004.docx]

Response to Reviewers

Manuscript Title:
“Prevalence, Acquisition, and Risk Factors of Carbapenem-Resistant Enterobacterales Colonization in Long-Term Care Hospitals: A Prospective Active Surveillance Study”

We sincerely thank the Editor and the Reviewers for their careful evaluation of our manuscript and for the constructive comments and suggestions. We have revised the manuscript accordingly and believe that these revisions have substantially improved the clarity and scientific rigor of the study. All changes in the revised manuscript are highlighted.

**Reviewer #1**

**Comment 1.**
What was the statistical method to calculate the sample size, or was it just based on those who gave consent? This method of sample size calculation may not reflect the actual number of colonizers in the facility.

Response:
We appreciate the reviewer’s insightful comment. This study was designed as a prospective exploratory active surveillance study rather than a prevalence estimation study based on formal sample size calculation. Enrollment depended on voluntary participation and written informed consent from eligible patients in the participating long-term care hospitals (LTCHs). We agree that this convenience sampling approach may not fully represent the actual burden of CRE colonization in the facilities. Therefore, we have clarified this point in the Methods and Limitations sections and acknowledged the potential for selection bias and limited generalizability.

**Comment 2**.
Please mention if the swabs were collected from patients once they were proved to be CRE positive. In other words how many patients were tested every week?

Response:
Thank you for this helpful comment. Once a patient was identified as CRE-positive during active surveillance, routine follow-up rectal swab cultures were not continued because the primary objective was to detect newly identified CRE colonization and acquisition among previously negative patients. Therefore, weekly surveillance cultures were subsequently performed only for patients who remained CRE-negative.

**Comment 3.**
Were these the same 140 patients remaining in the facility for six weeks? What about the newly admitted patients? Did that not change the sample size over six weeks?

Response:
We appreciate the reviewer’s comment. Not all enrolled participants completed the entire three month surveillance period because of discharge, death, or conversion to CRE-positive status. Newly admitted patients were enrolled continuously during the study period according to the predefined inclusion criteria. We have added clarification regarding follow-up duration and participant attrition in the revised manuscript.

**Comment 4.**
What was the status of symptomatic CRE colonizers within the study period?

Response:
This study primarily focused on asymptomatic CRE colonization detected through active surveillance cultures. Longitudinal clinical follow-up for symptomatic CRE infection was not systematically performed. However, the presence of clinical symptoms at the time of the initial surveillance culture was assessed and included in the analysis of risk factors for CRE acquisition. This limitation has been clarified in the revised manuscript.

**Comment 5.**
Table 1B. Explain how this was detected and its mechanism.

Response:
Thank you for this comment. We agree that the resistance mechanism should be described more clearly. In this study, carbapenemase production was assessed using the NG Test CARBA5 immunochromatographic assay. Among the 35 CRE isolates, 28 isolates (80.0%) were confirmed as carbapenemase-producing Enterobacterales (CPE). Of these, 27 isolates produced KPC and one isolate produced NDM. For the remaining seven non-CPE CRE isolates, no carbapenemase was detected by the CARBA5 assay. We have clarified the microbiological methods in the revised Methods section.

**Comment 6.**
Table 1B adds up to 35, but the previous table shows 34.

Response:
Thank you for identifying this discrepancy. Although 34 patients were CRE-positive, one patient simultaneously carried both Klebsiella pneumoniae and Escherichia coli, resulting in 35 CRE isolates. We have clarified this point in the Results section.

**Comment 7.**
What was the mechanism of non-CPE CRE? Was any attempt made to elicit the mechanism?

Response:
We appreciate the reviewer’s important comment. In this study, carbapenemase production was evaluated using the NG-Test CARBA5 assay. However, additional molecular investigations for non-carbapenemase resistance mechanisms, such as ESBL or AmpC production combined with porin loss, were not performed. Therefore, the precise resistance mechanisms of the non-CPE isolates could not be determined. We have acknowledged this limitation in the Discussion section.

**Comment 8.**
Antibiotic exposure… What was the status of current antibiotic therapy, if any?

Response:
Thank you for this valuable comment. Data regarding ongoing antibiotic therapy at the exact time of surveillance culture were incompletely available and therefore could not be systematically included in the final analysis. We agree that current antimicrobial exposure may influence CRE colonization and acquisition, and we have added this point as a limitation in the Discussion section.

**Comment 9.**
What was the incidence of colonization?

Response:
We appreciate the reviewer’s comment. The cumulative incidence of CRE acquisition among initially CRE-negative patients during the surveillance period was 8.6%. This information has been clarified in the revised Results and Discussion sections.

**Comment 10.**
Current antibiotic therapy also needs to be discussed as it would impact colonization of CRE.

Response:
We agree with the reviewer’s comment and have expanded the Discussion section to acknowledge that current antimicrobial therapy may influence both CRE colonization dynamics and acquisition risk. Current antibiotic therapy may substantially influence CRE colonization and acquisition by exerting selective antimicrobial pressure during hospitalization. In the present study, we analyzed prior antibiotic exposure within the previous three months; however, detailed longitudinal information regarding ongoing antibiotic therapy during the surveillance period was not systematically collected. Therefore, the potential impact of current antibiotic treatment on CRE acquisition could not be fully evaluated. We have added this point to the Discussion section as both an important consideration and a study limitation.

**Comment 11.**
This has not been captured in the patient selection criteria.

Response:
Thank you for this comment. We have revised the Methods section to provide clearer inclusion and exclusion criteria and clarified the definitions used for patient enrollment.

We agree that the description of “prior room sharing with CRE-positive patients” required further clarification. This variable was not part of the patient selection criteria but was collected as a clinical exposure variable during data extraction from medical and infection control records. Specifically, room-sharing exposure was defined as a history of sharing the same room with a patient known to have CRE colonization or infection within the previous year. We have clarified this definition in the Methods section of the revised manuscript.

**Comment 12.**
What sort of incentives do the researchers have in mind that may not have been unethical?

Response:
We appreciate the reviewer’s thoughtful comment. We agree that the wording regarding “incentives” may have been inappropriate or unclear. Therefore, we removed this expression from the manuscript and revised the sentence to indicate that recruitment was challenging because participation required repeated surveillance cultures and written informed consent.

**Comment 13.**
Were there any discharges from the study group within the six-week period? If so, how did it impact the outcome?

Response:
Thank you for this important comment. During the follow-up period, some patients were discharged or died before completion of the scheduled surveillance cultures. To appropriately account for these incomplete follow-up data, we additionally performed survival analysis using a Cox proportional hazards model incorporating both the date of CRE acquisition and censoring dates due to discharge or death during follow-up.

In the revised analysis, follow-up time was calculated from the date of the initial CRE-negative screening culture to the date of CRE acquisition or censoring. Patients who newly converted to CRE-positive status were considered to have experienced the study event on the date of CRE detection and were subsequently excluded from further surveillance. Patients who were discharged or died before CRE acquisition were treated as censored observations at their last available follow-up date.

After incorporating censoring information, the overall results were not substantially changed. Previous invasive device use within the past year remained significantly associated with CRE acquisition (HR 9.83, 95% CI 1.23–78.63, p=0.031), as did prior room sharing with a CRE-positive patient (HR 5.01, 95% CI 1.01–24.88, p=0.049). Therefore, we believe that discharge or death during follow-up did not materially alter the main findings of this study.

We have revised the Methods and Results sections accordingly and updated the Kaplan–Meier survival analysis and Cox proportional hazards analysis to reflect censoring during follow-up.

**Comment 14.**
Did co-morbidities of the patients impact colonization?

Response:
Underlying diseases were included as variables in the univariate Cox proportional-hazard analysis. However, no statistically significant association with CRE acquisition was observed in this cohort.

**Reviewer #2**

**Comment 1.**
Please specify the study design and study settings/centers.

Response:
Thank you for this comment. We have clarified that this study was a multicenter prospective observational active surveillance study conducted in four long-term care hospitals in Gyeongnam Province, Republic of Korea. In addition, we added a description of the role and function of Korean long-term care hospitals, which provide long-term medical and nursing care for elderly patients with chronic illnesses, functional dependency, and multiple comorbidities. However, we did not mention each hospital’s name specifically, because it would not be interesting for the readers.

**Comment 2.**
There is a discrepancy in the study timeline between the Abstract and Methods sections.

Response:
We appreciate the reviewer’s careful observation. The main reason of discrepancy in the study timeline was due to the total period of IRB permission and the real study period of recruitment of patients. The timeline discrepancy has been aligned, and the study period is now consistently stated throughout the manuscript.

**Comment 3.**
Inclusion and exclusion criteria need to be clearly defined.

Response:
We agree and have reorganized the Methods section to clearly present the inclusion and exclusion criteria.

**Comment 4.**
The sample size appears too small and may limit external validity.

Response:
We acknowledge this limitation. Because participation required informed consent and repeated surveillance cultures, the enrolled cohort represented a convenience sample rather than a statistically predefined population-based sample. We have explicitly acknowledged the limitation regarding sample size and generalizability in the revised manuscript.

**Comment 5.**
No multivariate analysis was performed.

Response:
We appreciate the reviewer’s important comment. Because the number of CRE acquisition events was limited, performing a stable multivariable Cox regression model was statistically difficult and could have resulted in model overfitting. Therefore, we performed exploratory univariate survival analyses to identify potential risk factors associated with CRE acquisition. This explanation has been added to the Statistical Analysis and Limitations sections.

**Comment 6.**
The inference regarding colonization at admission may be overinterpreted.

Response:
We agree with the reviewer and revised the wording accordingly. The statement now indicates that early detection of CRE “suggests that colonization may already have been present before or at the time of admission,” rather than definitively indicating acquisition status.

**Comment 7.**
The lack of genotyping and clonality experiments limits confirmation of intra-facility transmission.

Response:
We agree with the reviewer’s important point. We have strengthened the Limitation section by stating that molecular typing and clonality analyses were not performed; therefore, direct intra-facility transmission pathways and genetic relatedness among isolates could not be confirmed.

**Reviewer #3**

**Comment 1.**
Study design needs to be specified. Inclusion and exclusion criteria are not defined. How was the sample size of 140 determined?

Response:
We appreciate the reviewer’s comment. The study design and eligibility criteria have been clarified in the revised Methods section. We also clarified that enrollment was based on voluntary participation and informed consent rather than predefined statistical sample size estimation.

**Comment 2.**
Stress on how active surveillance can identify more colonization.

Response:
Thank you for this valuable suggestion. We have expanded the Discussion section to emphasize that active surveillance cultures can detect substantially more CRE colonization than symptom-based or clinical culture-based approaches alone.

**Comment 3.**
Was follow-up of infections performed for patients with CRE colonization?

Response:
The present study focused on asymptomatic CRE colonization detected through active surveillance cultures, and longitudinal follow-up for subsequent CRE infection was not systematically performed. This limitation has been clarified in the revised manuscript.

**Comment 4.**
Do the authors advocate routine screening?

Response:
We do not advocate universal routine screening in all LTCH settings. Rather, we believe that targeted surveillance of high-risk patients may represent a more feasible and cost-effective approach in resource-limited LTCH settings. This point has been clarified in the Discussion section.
