# Supplementary material for: Prevalence, acquisition, and risk factors of carbapenem-resistant enterobacterales colonization in long-term care hospitals: A prospective active surveillance study
Source: PLoS One. 2026 Sep 28;21(9):e0358973. doi: 10.1371/journal.pone.0358973 (PMC13618942; doi:10.1371/journal.pone.0358973)
Supplement: Peer review history

# Peer review: Decision Letter 0

**DOI:** 10.1371/journal.pone.0358973.r001

**Authors:** Iddya Karunasagar

5 May 2026

PONE-D-26-11890

Prevalence, Acquisition, and Risk Factors of Carbapenem-Resistant Enterobacterales Colonization in Long-Term Care Hospitals: A Prospective Active Surveillance Study

PLOS One

Dear Dr. Kim,

Thank you for submitting your manuscript to PLOS ONE. After careful consideration, we feel that it has merit but does not fully meet PLOS ONE’s publication criteria as it currently stands. Therefore, we invite you to submit a revised version of the manuscript that addresses the points raised during the review process.

==============================

Please see reviewer comments. There are questions about methodology and comments about inconsistencies in the data. Please revise addressing all comments and questions.

==============================

Please submit your revised manuscript by Jun 19 2026 11:59PM. If you will need more time than this to complete your revisions, please reply to this message or contact the journal office at plosone@plos.org. When you're ready to submit your revision, log on to https://www.editorialmanager.com/pone/ and select the 'Submissions Needing Revision' folder to locate your manuscript file.

Please include the following items when submitting your revised manuscript:

- A letter that responds to each point raised by the academic editor and reviewer(s). You should upload this letter as a separate file labeled 'Response to Reviewers'.
- A marked-up copy of your manuscript that highlights changes made to the original version. You should upload this as a separate file labeled 'Revised Manuscript with Track Changes'.
- An unmarked version of your revised paper without tracked changes. You should upload this as a separate file labeled 'Manuscript'.

If you would like to make changes to your financial disclosure, please include your updated statement in your cover letter. Guidelines for resubmitting your figure files are available below the reviewer comments at the end of this letter.

If applicable, we recommend that you deposit your laboratory protocols in protocols.io to enhance the reproducibility of your results. Protocols.io assigns your protocol its own identifier (DOI) so that it can be cited independently in the future. For instructions see: https://journals.plos.org/plosone/s/submission-guidelines#loc-laboratory-protocols. Additionally, PLOS ONE offers an option for publishing peer-reviewed Lab Protocol articles, which describe protocols hosted on protocols.io. Read more information on sharing protocols at https://plos.org/protocols?utm_medium=editorial-email&utm_source=authorletters&utm_campaign=protocols.

As the corresponding author, your ORCID iD is verified in the submission system and will appear in the published article. PLOS supports the use of ORCID, and we encourage all coauthors to register for an ORCID iD and use it as well. Please encourage your coauthors to verify their ORCID iD within the submission system before final acceptance, as unverified ORCID iDs will not appear in the published article. *Only*  the individual author can complete the verification step; PLOS staff *cannot*  verify ORCID iDs on behalf of authors.

We look forward to receiving your revised manuscript.

Kind regards,

Iddya Karunasagar

Academic Editor

PLOS One

**Journal Requirements:**

When submitting your revision, we need you to address these additional requirements.

1. Please ensure that your manuscript meets PLOS ONE's style requirements, including those for file naming. The PLOS ONE style templates can be found at

https://journals.plos.org/plosone/s/file?id=wjVg/PLOSOne_formatting_sample_main_body.pdf and

https://journals.plos.org/plosone/s/file?id=ba62/PLOSOne_formatting_sample_title_authors_affiliations.pdf

2. We note that the grant information you provided in the ‘Funding Information’ and ‘Financial Disclosure’ sections do not match.

When you resubmit, please ensure that you provide the correct grant numbers for the awards you received for your study in the ‘Funding Information’ section.

3. Please note that funding information should not appear in any section or other areas of your manuscript. We will only publish funding information present in the Funding Statement section of the online submission form. Please remove any funding-related text from the manuscript.

4. In the online submission form, you indicated that your data is available only on request from a third party. Please note that your Data Availability Statement is currently missing the contact details for the third party, such as an email address or a link to where data requests can be made. Please update your statement with the missing information.

5. Your ethics statement should only appear in the Methods section of your manuscript. If your ethics statement is written in any section besides the Methods, please move it to the Methods section and delete it from any other section. Please ensure that your ethics statement is included in your manuscript, as the ethics statement entered into the online submission form will not be published alongside your manuscript.

6. Please upload a new copy of Figures 1 and 2 as the detail is not clear. Please follow the link for more information:  https://journals.plos.org/plosone/s/figures

7. We notice that your supplementary tables are included in the manuscript file. Please remove them and upload them with the file type 'Supporting Information'. Please ensure that each Supporting Information file has a legend listed in the manuscript after the references list.

8. If the reviewer comments include a recommendation to cite specific previously published works, please review and evaluate these publications to determine whether they are relevant and should be cited. There is no requirement to cite these works unless the editor has indicated otherwise.

**Additional Editor Comments:**

Please see the reviewer comments. There are a number of comments and clarifications needed. Please revise addressing all comments and questions.

[Note: HTML markup is below. Please do not edit.]

Reviewers' comments:

Reviewer's Responses to Questions

**Comments to the Author**

1. Is the manuscript technically sound, and do the data support the conclusions?

The manuscript must describe a technically sound piece of scientific research with data that supports the conclusions. Experiments must have been conducted rigorously, with appropriate controls, replication, and sample sizes. The conclusions must be drawn appropriately based on the data presented.

Reviewer #1: Partly

Reviewer #2: Partly

Reviewer #3: Yes

**********

2. Has the statistical analysis been performed appropriately and rigorously?

Reviewer #1: Yes

Reviewer #2: No

Reviewer #3: Yes

**********

3. Have the authors made all data underlying the findings in their manuscript fully available?

The PLOS Data policy requires authors to make all data underlying the findings described in their manuscript fully available without restriction, with rare exception (please refer to the Data Availability Statement in the manuscript PDF file). The data should be provided as part of the manuscript or its supporting information, or deposited to a public repository. For example, in addition to summary statistics, the data points behind means, medians and variance measures should be available. If there are restrictions on publicly sharing data—e.g. participant privacy or use of data from a third party—those must be specified.

Reviewer #1: Yes

Reviewer #2: No

Reviewer #3: Yes

**********

4. Is the manuscript presented in an intelligible fashion and written in standard English?

PLOS ONE does not copyedit accepted manuscripts, so the language in submitted articles must be clear, correct, and unambiguous. Any typographical or grammatical errors should be corrected at revision, so please note any specific errors here.

Reviewer #1: Yes

Reviewer #2: Yes

Reviewer #3: Yes

**********

5. Review Comments to the Author

Please use the space provided to explain your answers to the questions above. You may also include additional comments for the author, including concerns about dual publication, research ethics, or publication ethics. (Please upload your review as an attachment if it exceeds 20,000 characters)

Reviewer #1: The authors have done a longitudinal cohort study to determine the acquisition of CRE Enterobacterales.

Two groups have been discussed. Long-term colonizers and fresh acquisitions that capture the pattern.

Specific comments pertaining to the study:

1.L-130 :What was the statistical method to calculate the sample size, or was it just based on those who gave consent? This method of sample size calculation may not reflect the actual number of colonizers in the facility.

2.L-107 :Please mention if the swabs were collected from patients once they were proved to be CRE positive. In other words how many patients were tested every week?

3.L- 108:Were these the same 140 patients remaining in the facility for six weeks??

What about the newly admitted patients ? Did that not change the sample size over six weeks?

4.L-139:What was the status of symptomatic CRE colonizers within the study period?

5.Table1 B. Explain how this was detected and its mechanism.

6..Table1- Adds up to 35. But your previous table shows 34.

7.Table 1-: B Non CPE: What was the mechanism of CRE? Was any attempt made to elicit the mechanism?

8.Table 2- Antibiotic exposure... What was the status of current antibiotic therapy, if any?

9.L-176:- What was the incidence of colonization ?

10.L-197 : Current antibiotic therapy also needs to be discussed as it would impact colonization of CRE.

11.L-231:This has not been captured in the patient selection criteria.

12.What sort of incentives do the researchers have in mind that may not have been unethical?

13.Were there any discharges from the study group within the six-week period? If so, how did it impact the outcome?

14. Did co-morbidities of the patients impact colonization?

Reviewer #2: This present study encompasses a crucial and extremely relevant issue by evaluating carbapenem-resistant Enterobacterales (CRE) colonization in long-term care hospitals. These hospitals are increasingly being recognized as key reservoirs for AMR. The forward-looking study design intended to focus on importation and in-facility acquisition providing valuable insights into transmission dynamics. However, the study has several major concerns, especially related to Methodology which if not addressed would lead to inappropriate interpretation of data.

METHODS

Enrollment of patients for active surveillance began on 14/11/2023, and continued through 31/12/2024 in four LTCHs in Gyeonggnam province, Korea.

Comment: Please specify the study design and study settings/centers. There is a clear discrepancy in the timeline of the study mentioned under ABSTRACT (September 2022 to December 2024) and METHODS section (began on 14/11/2023, and continued through

31/12/2024).

Both newly admitted patients (≤2 days after admission) and current inpatients (>2 days after admission) were eligible. Among 1,179 patients admitted during the study period, 26 patients with documented CRE colonization or infection within the previous 102 year were excluded.

Comment: Inclusion criteria and exclusion criteria need to be mentioned properly. Number of patients and number of patients excluded are essentially part of Descriptive Statistics and need to be mentioned under ‘Results’ section, not under ‘Methods’.

The categorization of cases into newly admitted patients and current inpatients are arbitrary, especially the current inpatients. The latter group may be heterogenous and may include patients who are admitted for long. So, there should be clear inclusion criteria at the very beginning of the study. Or, the admitted patients >2 days may be further stratified by duration of stay.

Of the remaining 1,153 eligible patients, 140 who provided informed consent were finally enrolled.

Comment: The sample size seems to low, approximately 12% of the total eligible percentage. Authors have not mentioned any desired sample size. A small sample size lacks external validity. The prevalence of CRE infection may not be appropriate.

In addition, to explore potential risk factors for CRE acquisition, a Cox proportional-hazard model was applied among patients who were CRE-negative at initial screening, using CRE acquisition events occurring during the follow-up period of up to 42 days.

Comment: It seems to be that only univariate analysis have been performed. No multivariate adjustment was not caried out to tackle confounder like Severity of illness, Length of stay, Antibiotic exposure. If doing multivariate analysis was not feasible. Kindly justify the same.

RESULTS

Most CRE-positive patients (70.6%) were detected as positive at the first surveillance test,

indicating colonization at or near the time of admission.

Comment: The above inference may not be accurate. The newly admitted patients may be colonized in their previous hospital. Therefore, without clear evidence of screened negative at previous hospital, there may be overinterpretation of rate of acquisition.

DISCUSSION

Molecular study may provide more concrete transmission or acquisition data.

Comment: The lack of genotyping and clonality experiments limits the confirmation of intra-facility transmission of in-hospital acquisition of CRE. Authors are advised to acknowledge this issue strongly under the Limitation section.

Reviewer #3: Study design needs to be specified . Inclusion and exclusion criteria are not defined.how is the sample size of 140 arrived at.

In discussion . stress on how active surveillance can pick on more colonization,

Was follow up of infections done for patients with CRE colonization? Do the authors advocate routine screening?

**********

6. PLOS authors have the option to publish the peer review history of their article (what does this mean?). If published, this will include your full peer review and any attached files.

If you choose “no”, your identity will remain anonymous but your review may still be made public.

**Do you want your identity to be public for this peer review?**  For information about this choice, including consent withdrawal, please see our Privacy Policy.

Reviewer #1: No

Reviewer #2: **Yes:** Ujjala Ghoshal

Reviewer #3: No

**********

[NOTE: If reviewer comments were submitted as an attachment file, they will be attached to this email and accessible via the submission site. Please log into your account, locate the manuscript record, and check for the action link "View Attachments". If this link does not appear, there are no attachment files.]

To ensure your figures meet our technical requirements, please review our figure guidelines: https://journals.plos.org/plosone/s/figures

You may also use PLOS’s free figure tool, NAAS, to help you prepare publication quality figures: https://journals.plos.org/plosone/s/figures#loc-tools-for-figure-preparation.

NAAS will assess whether your figures meet our technical requirements by comparing each figure against our figure specifications.

Attachment Submitted filename: CRE_LTCH_PLOS_ONE (submitted)-PLOS.docx

# Author comment: Author response to Decision Letter 1

**DOI:** 10.1371/journal.pone.0358973.r002

22 Jun 2026

Response to Reviewers

Manuscript Title:

“Prevalence, Acquisition, and Risk Factors of Carbapenem-Resistant Enterobacterales Colonization in Long-Term Care Hospitals: A Prospective Active Surveillance Study”

We sincerely thank the Editor and the Reviewers for their careful evaluation of our manuscript and for the constructive comments and suggestions. We have revised the manuscript accordingly and believe that these revisions have substantially improved the clarity and scientific rigor of the study. All changes in the revised manuscript are highlighted.

Reviewer #1

Comment 1.

What was the statistical method to calculate the sample size, or was it just based on those who gave consent? This method of sample size calculation may not reflect the actual number of colonizers in the facility.

Response:

We appreciate the reviewer’s insightful comment. This study was designed as a prospective exploratory active surveillance study rather than a prevalence estimation study based on formal sample size calculation. Enrollment depended on voluntary participation and written informed consent from eligible patients in the participating long-term care hospitals (LTCHs). We agree that this convenience sampling approach may not fully represent the actual burden of CRE colonization in the facilities. Therefore, we have clarified this point in the Methods and Limitations sections and acknowledged the potential for selection bias and limited generalizability.

Comment 2.

Please mention if the swabs were collected from patients once they were proved to be CRE positive. In other words how many patients were tested every week?

Response:

Thank you for this helpful comment. Once a patient was identified as CRE-positive during active surveillance, routine follow-up rectal swab cultures were not continued because the primary objective was to detect newly identified CRE colonization and acquisition among previously negative patients. Therefore, weekly surveillance cultures were subsequently performed only for patients who remained CRE-negative.

Comment 3.

Were these the same 140 patients remaining in the facility for six weeks? What about the newly admitted patients? Did that not change the sample size over six weeks?

Response:

We appreciate the reviewer’s comment. Not all enrolled participants completed the entire three month surveillance period because of discharge, death, or conversion to CRE-positive status. Newly admitted patients were enrolled continuously during the study period according to the predefined inclusion criteria. We have added clarification regarding follow-up duration and participant attrition in the revised manuscript.

Comment 4.

What was the status of symptomatic CRE colonizers within the study period?

Response:

This study primarily focused on asymptomatic CRE colonization detected through active surveillance cultures. Longitudinal clinical follow-up for symptomatic CRE infection was not systematically performed. However, the presence of clinical symptoms at the time of the initial surveillance culture was assessed and included in the analysis of risk factors for CRE acquisition. This limitation has been clarified in the revised manuscript.

Comment 5.

Table 1B. Explain how this was detected and its mechanism.

Response:

Thank you for this comment. We agree that the resistance mechanism should be described more clearly. In this study, carbapenemase production was assessed using the NG Test CARBA5 immunochromatographic assay. Among the 35 CRE isolates, 28 isolates (80.0%) were confirmed as carbapenemase-producing Enterobacterales (CPE). Of these, 27 isolates produced KPC and one isolate produced NDM. For the remaining seven non-CPE CRE isolates, no carbapenemase was detected by the CARBA5 assay. We have clarified the microbiological methods in the revised Methods section.

Comment 6.

Table 1B adds up to 35, but the previous table shows 34.

Response:

Thank you for identifying this discrepancy. Although 34 patients were CRE-positive, one patient simultaneously carried both Klebsiella pneumoniae and Escherichia coli, resulting in 35 CRE isolates. We have clarified this point in the Results section.

Comment 7.

What was the mechanism of non-CPE CRE? Was any attempt made to elicit the mechanism?

Response:

We appreciate the reviewer’s important comment. In this study, carbapenemase production was evaluated using the NG-Test CARBA5 assay. However, additional molecular investigations for non-carbapenemase resistance mechanisms, such as ESBL or AmpC production combined with porin loss, were not performed. Therefore, the precise resistance mechanisms of the non-CPE isolates could not be determined. We have acknowledged this limitation in the Discussion section.

Comment 8.

Antibiotic exposure… What was the status of current antibiotic therapy, if any?

Response:

Thank you for this valuable comment. Data regarding ongoing antibiotic therapy at the exact time of surveillance culture were incompletely available and therefore could not be systematically included in the final analysis. We agree that current antimicrobial exposure may influence CRE colonization and acquisition, and we have added this point as a limitation in the Discussion section.

Comment 9.

What was the incidence of colonization?

Response:

We appreciate the reviewer’s comment. The cumulative incidence of CRE acquisition among initially CRE-negative patients during the surveillance period was 8.6%. This information has been clarified in the revised Results and Discussion sections.

Comment 10.

Current antibiotic therapy also needs to be discussed as it would impact colonization of CRE.

Response:

We agree with the reviewer’s comment and have expanded the Discussion section to acknowledge that current antimicrobial therapy may influence both CRE colonization dynamics and acquisition risk. Current antibiotic therapy may substantially influence CRE colonization and acquisition by exerting selective antimicrobial pressure during hospitalization. In the present study, we analyzed prior antibiotic exposure within the previous three months; however, detailed longitudinal information regarding ongoing antibiotic therapy during the surveillance period was not systematically collected. Therefore, the potential impact of current antibiotic treatment on CRE acquisition could not be fully evaluated. We have added this point to the Discussion section as both an important consideration and a study limitation.

Comment 11.

This has not been captured in the patient selection criteria.

Response:

Thank you for this comment. We have revised the Methods section to provide clearer inclusion and exclusion criteria and clarified the definitions used for patient enrollment.

We agree that the description of “prior room sharing with CRE-positive patients” required further clarification. This variable was not part of the patient selection criteria but was collected as a clinical exposure variable during data extraction from medical and infection control records. Specifically, room-sharing exposure was defined as a history of sharing the same room with a patient known to have CRE colonization or infection within the previous year. We have clarified this definition in the Methods section of the revised manuscript.

Comment 12.

What sort of incentives do the researchers have in mind that may not have been unethical?

Response:

We appreciate the reviewer’s thoughtful comment. We agree that the wording regarding “incentives” may have been inappropriate or unclear. Therefore, we removed this expression from the manuscript and revised the sentence to indicate that recruitment was challenging because participation required repeated surveillance cultures and written informed consent.

Comment 13.

Were there any discharges from the study group within the six-week period? If so, how did it impact the outcome?

Response:

Thank you for this important comment. During the follow-up period, some patients were discharged or died before completion of the scheduled surveillance cultures. To appropriately account for these incomplete follow-up data, we additionally performed survival analysis using a Cox proportional hazards model incorporating both the date of CRE acquisition and censoring dates due to discharge or death during follow-up.

In the revised analysis, follow-up time was calculated from the date of the initial CRE-negative screening culture to the date of CRE acquisition or censoring. Patients who newly converted to CRE-positive status were considered to have experienced the study event on the date of CRE detection and were subsequently excluded from further surveillance. Patients who were discharged or died before CRE acquisition were treated as censored observations at their last available follow-up date.

After incorporating censoring information, the overall results were not substantially changed. Previous invasive device use within the past year remained significantly associated with CRE acquisition (HR 9.83, 95% CI 1.23–78.63, p=0.031), as did prior room sharing with a CRE-positive patient (HR 5.01, 95% CI 1.01–24.88, p=0.049). Therefore, we believe that discharge or death during follow-up did not materially alter the main findings of this study.

We have revised the Methods and Results sections accordingly and updated the Kaplan–Meier survival analysis and Cox proportional hazards analysis to reflect censoring during follow-up.

Comment 14.

Did co-morbidities of the patients impact colonization?

Response:

Underlying diseases were included as variables in the univariate Cox proportional-hazard analysis. However, no statistically significant association with CRE acquisition was observed in this cohort.

Reviewer #2

Comment 1.

Please specify the study design and study settings/centers.

Response:

Thank you for this comment. We have clarified that this study was a multicenter prospective observational active surveillance study conducted in four long-term care hospitals in Gyeongnam Province, Republic of Korea. In addition, we added a description of the role and function of Korean long-term care hospitals, which provide long-term medical and nursing care for elderly patients with chronic illnesses, functional dependency, and multiple comorbidities. However, we did not mention each hospital’s name specifically, because it would not be interesting for the readers.

Comment 2.

There is a discrepancy in the study timeline between the Abstract and Methods sections.

Response:

We appreciate the reviewer’s careful observation. The main reason of discrepancy in the study timeline was due to the total period of IRB permission and the real study period of recruitment of patients. The timeline discrepancy has been aligned, and the study period is now consistently stated throughout the manuscript.

Comment 3.

Inclusion and exclusion criteria need to be clearly defined.

Response:

We agree and have reorganized the Methods section to clearly present the inclusion and exclusion criteria.

Comment 4.

The sample size appears too small and may limit external validity.

Response:

We acknowledge this limitation. Because participation required informed consent and repeated surveillance cultures, the enrolled cohort represented a convenience sample rather than a statistically predefined population-based sample. We have explicitly acknowledged the limitation regarding sample size and generalizability in the revised manuscript.

Comment 5.

No multivariate analysis was performed.

Response:

We appreciate the reviewer’s important comment. Because the number of CRE acquisition events was limited, performing a stable multivariable Cox regression model was statistically difficult and could have resulted in model overfitting. Therefore, we performed exploratory univariate survival analyses to identify potential risk factors associated with CRE acquisition. This explanation has been added to the Statistical Analysis and Limitations sections.

Comment 6.

The inference regarding colonization at admission may be overinterpreted.

Response:

We agree with the reviewer and revised the wording accordingly. The statement now indicates that early detection of CRE “suggests that colonization may already have been present before or at the time of admission,” rather than definitively indicating acquisition status.

Comment 7.

The lack of genotyping and clonality experiments limits confirmation of intra-facility transmission.

Response:

We agree with the reviewer’s important point. We have strengthened the Limitation section by stating that molecular typing and clonality analyses were not performed; therefore, direct intra-facility transmission pathways and genetic relatedness among isolates could not be confirmed.

Reviewer #3

Comment 1.

Study design needs to be specified. Inclusion and exclusion criteria are not defined. How was the sample size of 140 determined?

Response:

We appreciate the reviewer’s comment. The study design and eligibility criteria have been clarified in the revised Methods section. We also clarified that enrollment was based on voluntary participation and informed consent rather than predefined statistical sample size estimation.

Comment 2.

Stress on how active surveillance can identify more colonization.

Response:

Thank you for this valuable suggestion. We have expanded the Discussion section to emphasize that active surveillance cultures can detect substantially more CRE colonization than symptom-based or clinical culture-based approaches alone.

Comment 3.

Was follow-up of infections performed for patients with CRE colonization?

Response:

The present study focused on asymptomatic CRE colonization detected through active surveillance cultures, and longitudinal follow-up for subsequent CRE infection was not systematically performed. This limitation has been clarified in the revised manuscript.

Comment 4.

Do the authors advocate routine screening?

Response:

We do not advocate universal routine screening in all LTCH settings. Rather, we believe that targeted surveillance of high-risk patients may represent a more feasible and cost-effective approach in resource-limited LTCH settings. This point has been clarified in the Discussion section.

Attachment Submitted filename: Response to Reviewers.docx

# Peer review: Decision Letter 1

**DOI:** 10.1371/journal.pone.0358973.r003

**Authors:** Iddya Karunasagar

8 Sep 2026

Prevalence, Acquisition, and Risk Factors of Carbapenem-Resistant Enterobacterales Colonization in Long-Term Care Hospitals: A Prospective Active Surveillance Study

PONE-D-26-11890R1

Dear Dr. Kim,

We’re pleased to inform you that your manuscript has been judged scientifically suitable for publication and will be formally accepted for publication once it meets all outstanding technical requirements.

Within one week, you’ll receive an e-mail detailing the required amendments. When these have been addressed, you’ll receive a formal acceptance letter and your manuscript will be scheduled for publication.

An invoice will be generated when your article is formally accepted. Please note, if your institution has a publishing partnership with PLOS and your article meets the relevant criteria, all or part of your publication costs will be covered. Please make sure your user information is up-to-date by logging into Editorial Manager at Editorial Manager® and clicking the ‘Update My Information' link at the top of the page. For questions related to billing, please contact billing support.

If your institution or institutions have a press office, please notify them about your upcoming paper to help maximize its impact. If they’ll be preparing press materials, please inform our press team as soon as possible -- no later than 48 hours after receiving the formal acceptance. Your manuscript will remain under strict press embargo until 2 pm Eastern Time on the date of publication. For more information, please contact onepress@plos.org.

Kind regards,

Iddya Karunasagar

Academic Editor

PLOS One

Additional Editor Comments (optional):

All reviewer comments have been addressed.

Reviewers' comments:

Reviewer's Responses to Questions

**Comments to the Author**

Reviewer #1: All comments have been addressed

**********

2. Is the manuscript technically sound, and do the data support the conclusions?

Reviewer #1: Yes

**********

3. Has the statistical analysis been performed appropriately and rigorously?

Reviewer #1: Yes

**********

4. Have the authors made all data underlying the findings in their manuscript fully available?

The PLOS Data policy

Reviewer #1: Yes

**********

5. Is the manuscript presented in an intelligible fashion and written in standard English?

Reviewer #1: Yes

**********

Reviewer #1: (No Response)

**********

what does this mean?). If published, this will include your full peer review and any attached files.

If you choose “no”, your identity will remain anonymous but your review may still be made public.

**Do you want your identity to be public for this peer review?**  For information about this choice, including consent withdrawal, please see our Privacy Policy

Reviewer #1: No

**********

# Editor report: Acceptance letter

**DOI:** 10.1371/journal.pone.0358973.r004

**Authors:** Iddya Karunasagar

PONE-D-26-11890R1

PLOS One

Dear Dr. Kim,

I'm pleased to inform you that your manuscript has been deemed suitable for publication in PLOS One. Congratulations! Your manuscript is now being handed over to our production team.

At this stage, our production department will prepare your paper for publication. This includes ensuring the following:

* All references, tables, and figures are properly cited

* All relevant supporting information is included in the manuscript submission,

* There are no issues that prevent the paper from being properly typeset

You will receive further instructions from the production team, including instructions on how to review your proof when it is ready. Please keep in mind that we are working through a large volume of accepted articles, so please give us a few days to review your paper and let you know the next and final steps.

Lastly, if your institution or institutions have a press office, please let them know about your upcoming paper now to help maximize its impact. If they'll be preparing press materials, please inform our press team within the next 48 hours. Your manuscript will remain under strict press embargo until 2 pm Eastern Time on the date of publication. For more information, please contact onepress@plos.org.

You will receive an invoice from PLOS for your publication fee after your manuscript has reached the completed accept phase. If you receive an email requesting payment before acceptance or for any other service, this may be a phishing scheme. Learn how to identify phishing emails and protect your accounts at https://explore.plos.org/phishing.

If we can help with anything else, please email us at customercare@plos.org.

Thank you for submitting your work to PLOS One and supporting open access.

Kind regards,

PLOS One Editorial Office Staff

on behalf of

Dr. Iddya Karunasagar

Academic Editor

PLOS One
